# Alignment of Breathing Metal–Organic Framework Particles for Enhanced Water-Driven Actuation

**DOI:** 10.1021/acs.chemmater.3c01186

**Published:** 2023-08-25

**Authors:** Jacopo Andreo, Alejandra Durán Balsa, Min Ying Tsang, Anna Sinelshchikova, Orysia Zaremba, Stefan Wuttke, Jia Min Chin

**Affiliations:** † BCMaterials, Basque Center for Materials, Applications and Nanostructures, UPV/EHU Science Park, Leioa 48940, Spain; ‡ Faculty of Chemistry, Department of Functional Materials and Catalysis, University of Vienna, Währingerstr. 42, Vienna A-1090, Austria; § Ikerbasque, Basque Foundation for Science, Bilbao 48009, Spain

## Abstract

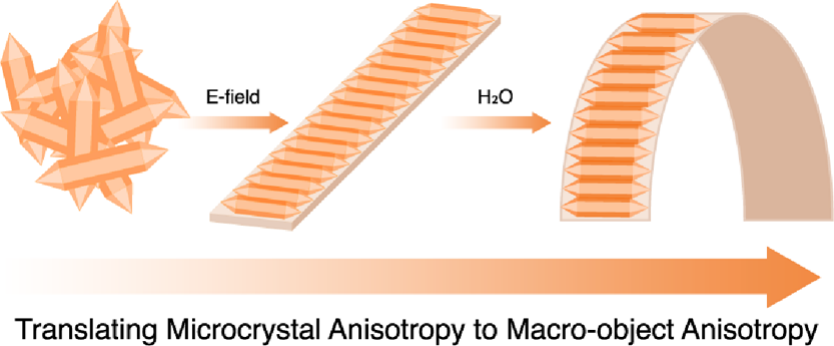

As the majority of known metal–organic frameworks (MOFs) possess anisotropic crystal lattices and thus anisotropic physicochemical properties, a pressing practical challenge in MOF research is the establishment of robust and simple processing methods to fully harness the anisotropic properties of the MOFs in various applications. We address this challenge by applying an E-field to precisely align MIL-88A microcrystals and generate MIL-88A@polymer films. Thereafter, we demonstrate the impact of MOF crystal alignment on the actuation properties of the films as a proof of concept. We investigate how different anisotropies of the MIL-88A@polymer films, specifically, crystal anisotropy, particle alignment, and film composition, can lead to the synergetic enhancement of the film actuation upon water exposure. Moreover, we explore how the directionality in application of the external stimuli (dry/humid air stream, water/air interface) affects the direction and the extent of the MIL-88A@polymer film movement. Apart from the superior water-driven actuation properties of the developed films, we demonstrate by dynamometer measurements the higher degree of mechanical work performed by the aligned MIL-88A@polymer films with the preserved anisotropies compared to the unaligned films. The insights provided by this work into anisotropic properties displayed by aligned MIL-88A@polymer films promise to translate crystal performance benefits measured in laboratories into real-world applications. We anticipate that our work is a starting point to utilize the full potential of anisotropic properties of MOFs.

## Introduction

Metal–organic frameworks (MOFs) have garnered significant attention as highly versatile materials, with reticular chemistry opening avenues to tailor their properties and functionality for various applications, ranging from gas storage and separation to catalysis and drug delivery.^1−3^ However, most MOFs bear non-cubic lattices, resulting in their properties and functionalities being highly anisotropic and dependent upon the crystallographic direction.^4,5^ For example, MOFs such as MIL-53 and MIL-68 and NU-1000 possess 1D channels along their long axis, affording higher molecular transport along that direction, while NU-1000 further displays significantly higher redox conductivity along its *c*-axis compared to that in the perpendicular direction.^6−8^ As such, to effectively design smart and efficient MOF-based materials that fully exploit their functional properties, the inherent anisotropy of most MOFs must be taken into account. Nevertheless, translation of such anisotropic properties of single MOF particles to useful macro-objects remains an outstanding challenge.^9,10^

Controlled alignment of MOF crystals is, therefore, a key focus to harnessing and translating the directional functionality of individual crystals to the macroscale;^11−13^ this can be achieved through various strategies, such as entropically driven or modulator-driven MOF ordering, *in situ* solvothermal growth on substrates that favor directional crystal growth, or secondary growth crystallization, or via external stimuli to obtain superlattices or directionally aligned MOF materials.^11,14−16^ Many of these methods require substrates, which hinder applicability, with few works utilizing these approaches to fabricate free-standing films.^17−19^ Furthermore, entropically driven assembly is often limited by demanding requirements such as a high degree of MOF crystal uniformity in terms of size and shape, while *in situ* growth may require carefully controlled synthetic conditions specifically tailored to the MOF of interest, which can be time-consuming to optimize. A new way to align MOF particles is via an electric-field (E-field)-assisted assembly. This strategy is based on exposure of a MOF dispersion to an E-field, resulting in induced polarization of the particles and their orientation along the direction of the E-field, allowing for dynamic alignment without the limitations encountered in entropically driven self-assembly.^20^ E-field alignment has proven to be a powerful tool to achieve large areas of oriented MOF particles with a high degree of control and reproducibility.^21^ Moreover, in this method, the formed MOF assembly can be fixed by a polymer matrix, thereby allowing the straightforward fabrication of ordered, large, and flexible MOF composite films.

To investigate the increased performance arising from harnessing the inherent anisotropy of MOFs, we turned to a MOF@polymer system: humidity-responsive MIL-88A@polymer actuator films. The addition of a polymer matrix to MOFs aids in their processing and shaping, thereby easing MOF integration into various technologies to advance their applications.^9^ Incorporating responsive domains such as breathing MOFs into synthetic matrices has garnered attention to generate materials that can respond to external stimuli (e.g., solvent, light, electricity, temperature)^22−26^ for applications in soft robotics, encapsulation, and sensing, among others.^27−30^

The well-studied Fe(III)-fumarate MOF, MIL-88A, exhibits solvent-responsive anisotropic breathing properties due to soft coordination bonds and flexible organic linkers.^31−34^ Férey et al. demonstrated through a combination of computational and powder X-ray diffraction that MIL-88A shows a decrease of the crystal *c* parameter, from 15.31 to 12.66 Å, and a concomitant increase in the *a* parameter, from 9.26 to 13.87 Å, when going from the post-synthetically dried form to the hydrated form.^35^ To fully exploit the strongly anisotropic breathing effect of MIL-88A for actuation, the crystals must be aligned relative to each other, because when the particles are randomly oriented, their swelling/shrinkage in multiple directions inevitably cancels out to some extent and results in a lower total directional swelling/shrinkage than when they are aligned. However, in currently reported MIL-88A composites, the MIL-88A particles are not uniformly oriented, hindering the propagation of the crystal swelling anisotropy to the macroscale. Maspoch et al. have shown humidity-driven self-folding of MIL-88A@PVDF films, whereby they use polydisperse MIL-88A particles to create a vertical gradient distribution throughout the film as well as HCl etching to design passive domains and obtain 3D architectures.^36,37^ He et al. also exploited the swelling properties of MIL-88A in a thermoplastic polyurethane film, which shows an actuation response to an increase in relative humidity (RH).^38^ Lastly, Hao et al. layered MIL-88A and silica particles in PVDF to obtain a photonic film after etching the silica particles.^39^ Various iterations of the MIL88-A polymer films have been reported and show humidity-driven actuation; however, they often require a high MOF loading of 50 wt % or more and post-synthetic modifications to achieve significant actuation.

In contrast to these works, we fabricate a MIL-88A@PEGDA film with significantly lower MOF loading (18 wt %) in which highly monodisperse MIL-88A particles are uniformly aligned. This alignment allows macroscale propagation of the particles’ inherently anisotropic swelling properties almost to the theoretical limit, which results in enhanced actuation upon exposure to water. By measuring the force of actuation of the aligned films, we further prove the importance of directional alignment. Furthermore, the choice of a flexible hydrophilic polymer—polyethylene glycol diacrylate (PEGDA)—as a film matrix leads to faster and more pronounced film movements as well as faster adsorption/desorption^40^ cycles without the need of high-temperature activation after adsorption, which overall strongly improves the direct applicability of the developed MIL-88A/polymer films.

## Results

### Particle Alignment

**Figure 1:**
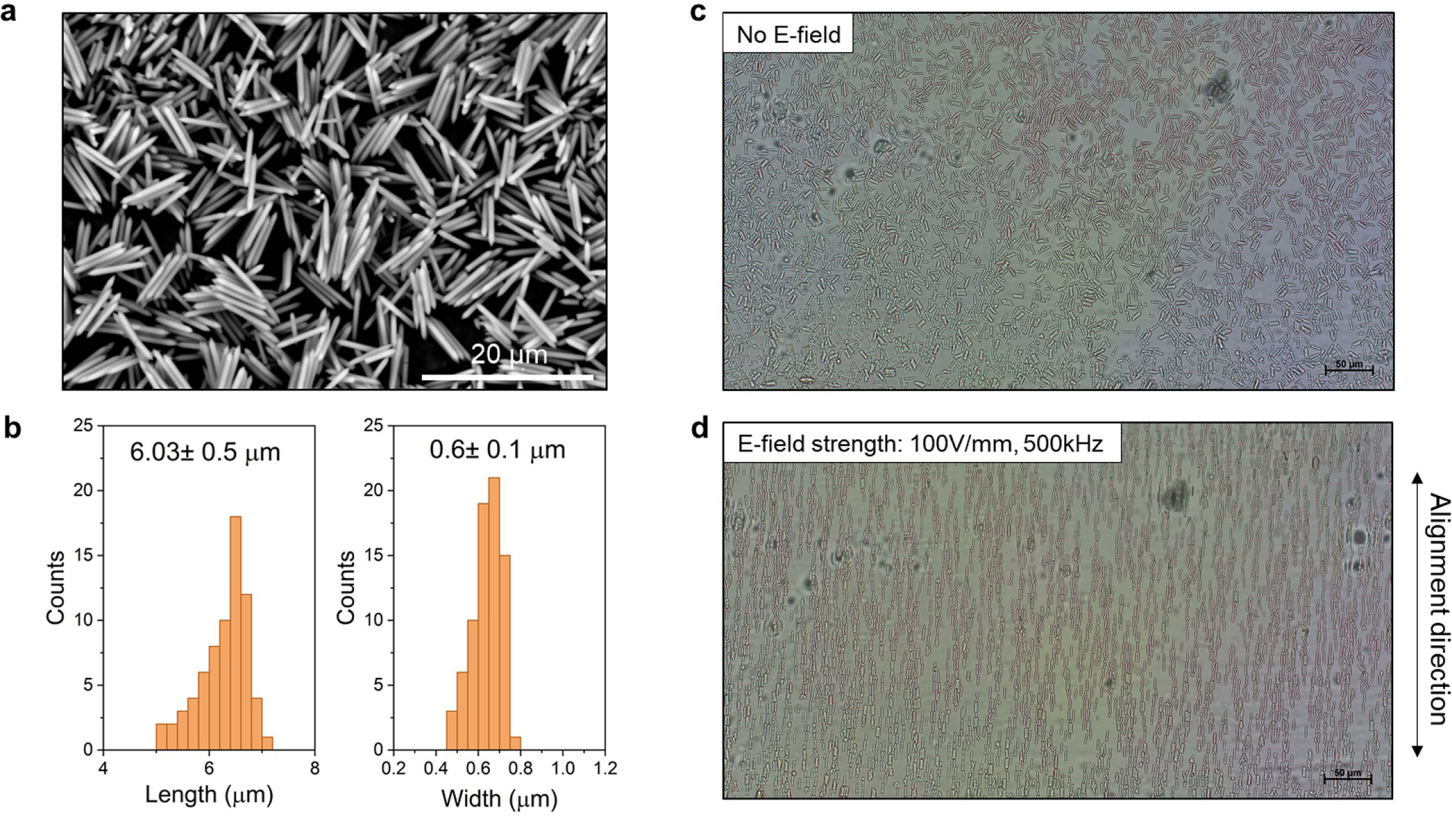
(a) SEM image of the as-synthesized MIL-88A rod particles. (b) Histograms of the particle length and width distribution. (c) Dispersion of the MIL-88A particle in DMA. (d) Oriented alignment of MIL-88A in DMA under E-field.

Monodisperse MIL-88A rod-shape crystals were synthesized via a surfactant-modulated reaction with FeCl_3_·6H_2_O and fumaric acid^41^ with average length 6.3 ± 0.5 μm and average width 0.6 ± 0.1 μm (SI 1.2, Figure 1a,b). The dried MIL-88A corresponds to the closed form and the wet MIL-88A corresponds to the open form of the structure. This was confirmed by PXRD patterns that are identical to the simulated patterns (SI 2.5, Figure S9). The optimal alignment parameters (solvent and frequency) were first determined for the as-synthesized MIL-88A crystals (SI 2.3). An efficient alignment response was found when using *N*,*N*-dimethylacetamide solvent (dielectric constant, ε = 37.8) and at 500 kHz. Upon applying the alternating current (AC) to generate an E-field, immediate particle alignment along the E-field direction was observed, followed by particle chaining (Figure 1d). The oriented alignment with the established conditions was repeated in the presence of PEGDA with average Mn = 250 and 700 (PEGDA_250_ and PEGDA_700_) and trimethylolpropane triacrylate (TMPTA) oligomer mixtures that were used for the film fabrication, and particle alignment was confirmed via optical microscopy. The particles aligned at a slower rate, and this can be explained by the higher viscosity of the oligomer mixture compared to DMA.

### MIL-88A@PEGDA Polymer Films

MIL-88A@PEGDA polymer films were prepared by dispersing as-synthesized MIL-88A (18 wt %) in a mixture of 9:9:1 ratio of PEGDA_700_, PEGDA_250_, and TMPTA with a 0.5 wt % TPO photoinitiator to obtain flexible films (SI 2.4). Two types of films (small and large) were fabricated to study the influence of aligned versus unaligned MIL-88A particles (SI 2.4, Figure S7). Homogeneously distributed films and films with a vertical gradient of particles were obtained by photopolymerization of the MOF/oligomer dispersion after 15 min and 1 h sedimentation under E-field, respectively.

**Figure 2:**
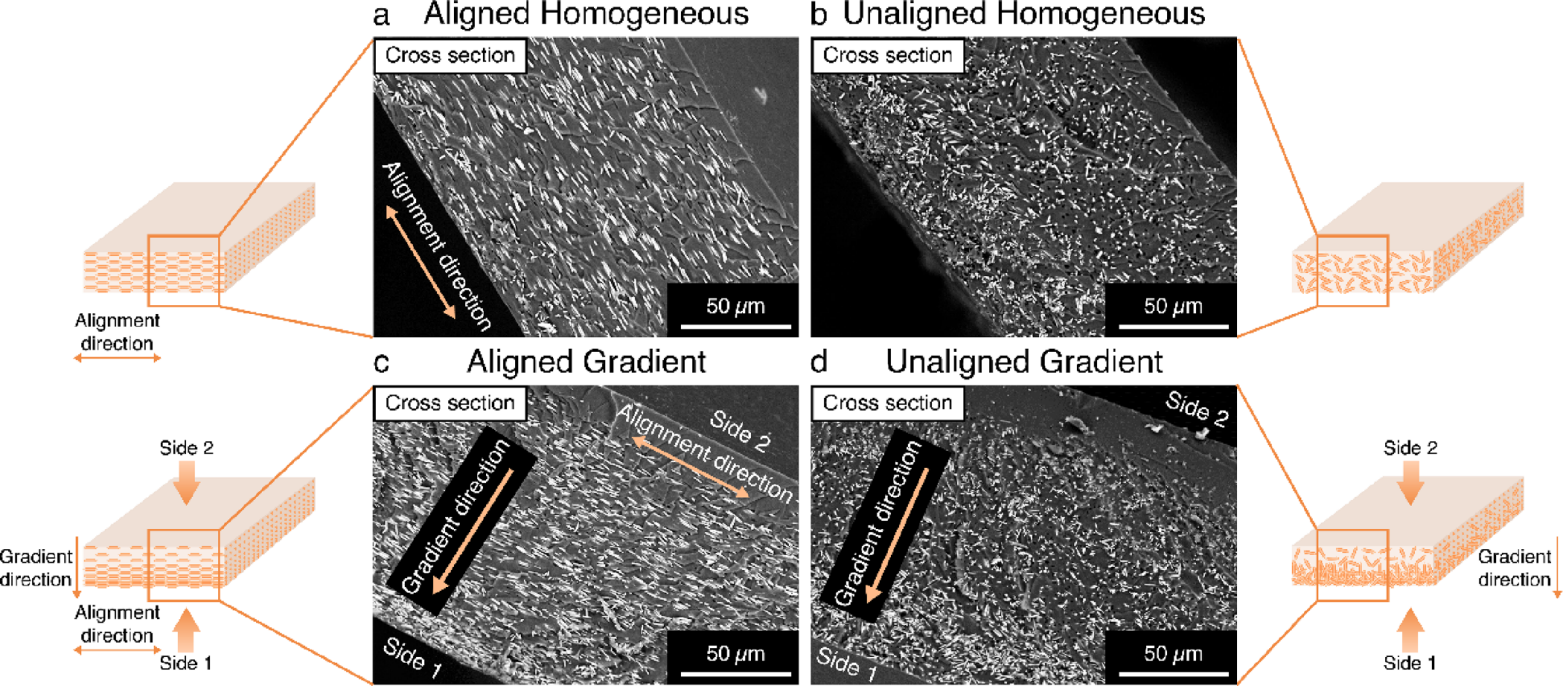
Selected SEM images of (a) aligned and (b) unaligned films with homogeneous distribution of MIL-88A and (c) aligned and (d) unaligned gradient distribution of MIL-88A particles with magnification 800× (more SEM images in SI 2.5).

Cross-section analysis of homogeneously distributed and gradient films by scanning electron microscopy (SEM) (Figure 2) confirmed the aligned orientation of MIL-88A particles in the PEGDA polymer films (SI 2.5, small film: Figure S10; large film: Figure S11). The SEM images (Figure 2) show particles aligned along the short side of the film (plane of the cross section), while for the unaligned MIL-88A@PEGDA films, particles are randomly oriented indicating no alignment. For both aligned and unaligned homogeneous films, the particles are distributed throughout the thickness evenly (Figure 2a,b) whereas for the gradient films, the MOFs are concentrated at the bottom, affording a vertical distribution gradient (Figure 2c,d). The aligned film appeared lighter in color than the unaligned film, which can be attributed to less obstruction of light passing through the ordered layer of MOFs versus the unordered MOF layer in the unaligned film (SI 2.4, Figure S6). The obtained films were then washed with EtOH, and a clear difference in folding was observed after washing; the aligned films showed a higher degree of curvature compared to the films without alignment (SI 2.4, Figure S7). Comprehensive characterization by powder X-ray diffraction (PXRD), Fourier transform infrared (FTIR) spectroscopy, and thermogravimetric analysis (TGA) of MIL-88A and the corresponding films was conducted, confirming the retained structure of MIL-88A and the composition of the MIL-88A@PEGDA films (SI 2.5).

### Actuation Properties

In our MIL-88A@PEGDA films, the expansion of the MIL-88A particles upon water adsorption drives the folding (actuation) of the film. The preliminary curvature of a series of small films with size ca. 3 × 21 × 0.1 mm (aligned–homogeneous, unaligned–homogeneous, aligned–gradient, and unaligned–gradient) and large films with size ca. 5 × 38 × 0.2 mm (aligned–homogeneous, unaligned–homogeneous, aligned–gradient, and unaligned–gradient) was compared when subjected to homogeneous changes in atmospheric humidity and complete immersion of the films in water. In general, the degree of curvature upon exposure to water is as follows: aligned–gradient > unaligned–gradient ∼ aligned–homogeneous > unaligned–homogeneous (SI 3.1, Table S2). The aligned films submerged in water curled more, faster, and more reliably than the unaligned ones. The unaligned films with homogeneous distribution of particles showed small or negligible movement once submerged.

As the volume of MIL-88A increased upon the opening of its pores, the change in thickness of the films was evaluated between dry (in the presence of silica gel under mild vacuum), atmospheric, and submerged conditions. Between dry and atmospheric humidity (60%), the change resulted in a 5.4% expansion for aligned films and a 3.7% expansion for the unaligned ones while the expansion between dry and immersed films in water was 12.4 and 11.3% for the aligned and unaligned films respectively (SI 3.1, Table S3).

**Figure 3:**
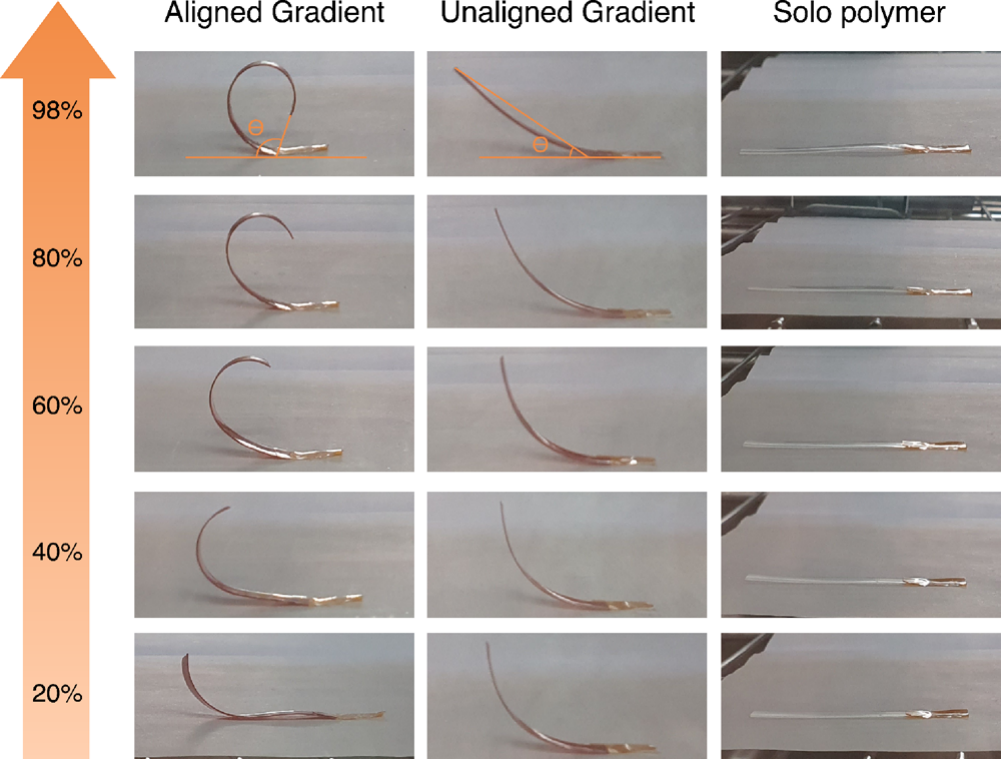
Actuation behavior of the large films (aligned–gradient and unaligned–gradient) and polymer at different humidity levels (20–98%).

The curvature response of the aligned and unaligned films was further tested with controlled humidity in a humidity chamber. The aligned-gradient large film showed an increased degree of curvature with increased humidity and vice versa, while the unaligned-gradient large film showed inconsistent curling behavior (Figure 3, details in SI 3.2, Figures S15 and S16). Additionally, the degree of curling in the aligned-gradient large film showed a linear relation with the change in humidity (SI 3.2, Figure S17).

**Figure 4:**
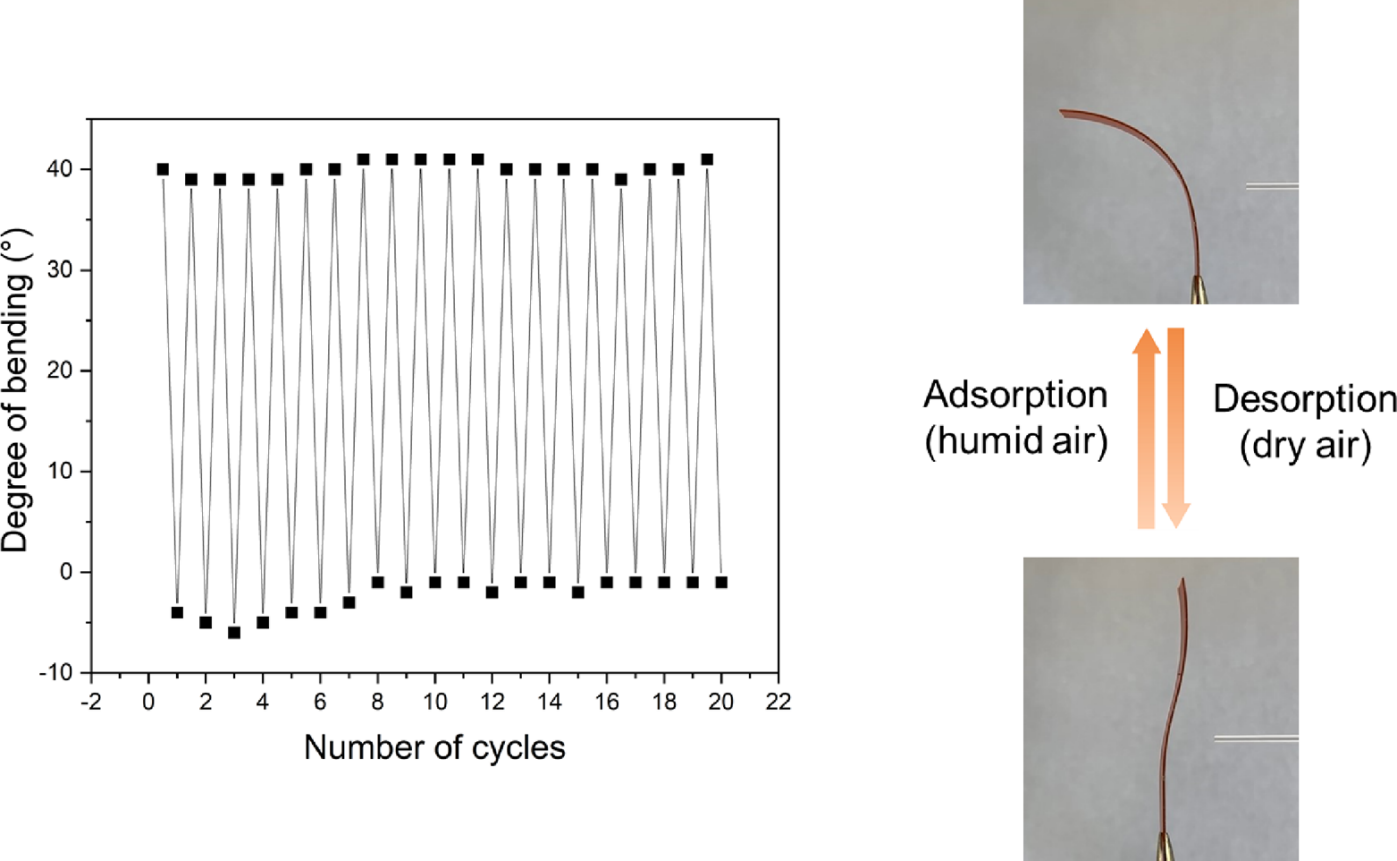
Cyclability of the aligned-gradient large film upon exposure to humid/dry air.

Our attention was then shifted to non-uniform interactions of the films with humid air and water. We employed two different techniques in this regard, either placing the films on the surface of water, where surface tension was sufficient to hold the composite in place, or using a gentle flow of humid (95% RH) or dry air directed perpendicularly to the film through a small nozzle (SI 1.10, Figure S4, SI 3.1, SI Videos 1–4). Under these directional stimuli, all MIL-88A@PEGDA polymer films changed shape readily but the curvature did not always occur in the same direction as in homogeneous conditions. A directional movement depending on the particle gradient was observed (SI 3.1, SI Videos 1–4). When the MOF-rich side (bottom of the fabricated film, side 1) was directionally exposed to water or humid air, the film readily curved along the longer axis away from the water source to a greater extent than upon exposure to humidity or water (see comparison in SI 3.1 and SI 3.3). The polymer-rich side (top of the film, side 2), on the other hand, showed a preferential movement along the short axis. This movement triggers a secondary movement along the long axis, which undergoes noticeable straightening. The dependence on the particle gradient of these movements was confirmed by the behavior of the homogeneous MIL-88A@PEGDA films, which showed the same directional movement on both faces, always preferentially curving along the long axis away from the water source. Films with unaligned particles showed similar movements to their aligned counterparts but were less significant and slower. Next, a flow of humid or dry air directed perpendicularly to the film through a small nozzle was tested. Upon exposing the films to humid air, the film curved along the long axis away from the water source, while in dry air flow, contrary movements were observed. The film movement induced by alternating humid and dry air was repeated for 20 cycles (each cycle included 1 min humid, 1 min dry), with negligible deviation in the film movement (Figure 4).

**Figure 5:**
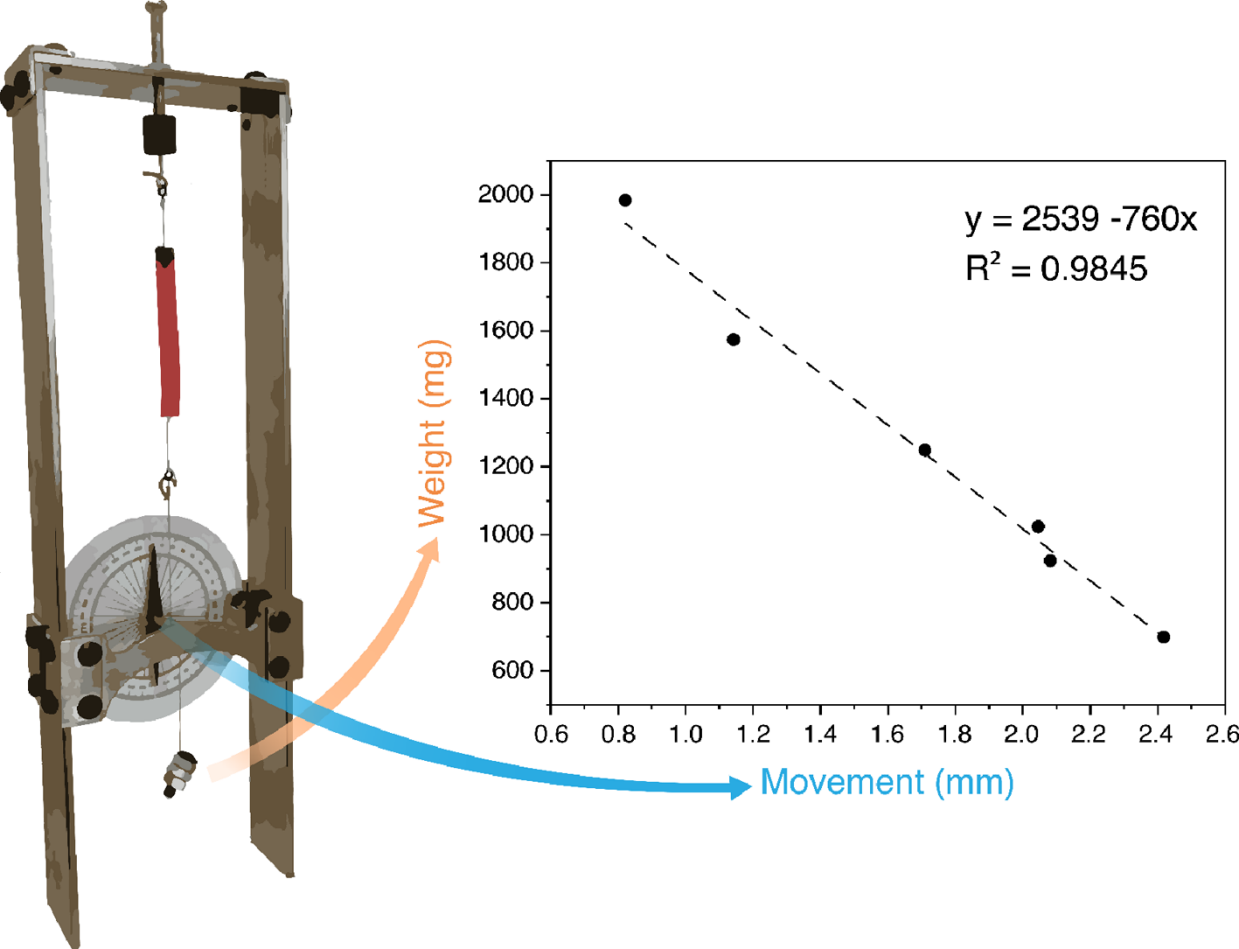
Mechanical strength of the aligned-gradient large films measured by a self-constructed dynamometer.

In order to evaluate the mechanical strength of this actuation behavior, we employed a self-constructed dynamometer (SI 1.8, Figure S2), designed based on mechanical hygrometers that convert the movement of a hair in angular movement against a calibrated weight.^42^ Similarly, we devised a device that would allow us to take an angular reading of the film’s overall change in length (due to curvature) against a set weight. By using a series of different weights, it was possible to extrapolate the general trend in mechanical strength of the film (Figure 5). In this experiment, the aligned-gradient large film (70 mg) lifted weights ranging from 700 to 1985 mg for a distance between 2.4 and 0.8 mm, resulting in a mean energy output of 18.1 ± 2.0 μJ. Movement within reliable resolution of the instrument was not detected for unaligned-gradient large films. Calibration of the instrument and data treatment can be found in SI 1.9 and SI 5 and Table S1.

## Discussion

The breathing behavior of MIL-88A makes it one of the ideal candidates to use in stimuli-responsive actuators. Unlike polydisperse MIL-88A particles used in previous reports to obtain a vertical gradient,^36,37^ the monodisperse MIL-88A particles used in this study have the advantage of uniform behavior. The microscopic images show that the dimensions (height H × width W) of a MIL-88A particle change from H 7.03 × W 1.3 μm (dried, closed form) to H 6.15 × W 1.72 μm (wet, open form), leading to ca. 32% increase in width and 14% decrease in height, concurrent with previous reports^37^ (SI 2.5, Figure S8). Swelling of MIL-88A is a strongly anisotropic process, whereby the MOF crystals shrink along the *c*-axis during concomitant expansion along the *a*/*b* axes. The aligned arrangement of these particles allows for their cumulative anisotropic swelling, leading to a higher overall film expansion. In order to achieve the directional orientation of the MIL-88A particles, an AC was used to generate an E-field, where the particles aligned along the direction of the E field. *N*,*N*-Dimethylacetamide was used as the solvent because it allowed for well-dispersed particles, and the high dielectric constant (ε =37.8) screened the MIL-88A intermolecular repulsions; thus, directional orientation and chaining could be observed. Particles can orient in an E-field via different mechanisms. Under the E-field at low frequencies (ca. 1 kHz), the predominant mechanism is polarization of the electronic double layer surrounding the particle. However, ions throughout the media also respond to lower frequencies, creating electroosmotic flows that can disrupt the alignment of the MOF particles.^21^ At high frequencies (ca. 500 kHz), used in this work, particles predominantly align via dielectric polarization of the particle itself,^43^ but the polarization of the electronic double layer may still contribute to the orientation of the particles.

**Figure 6:**
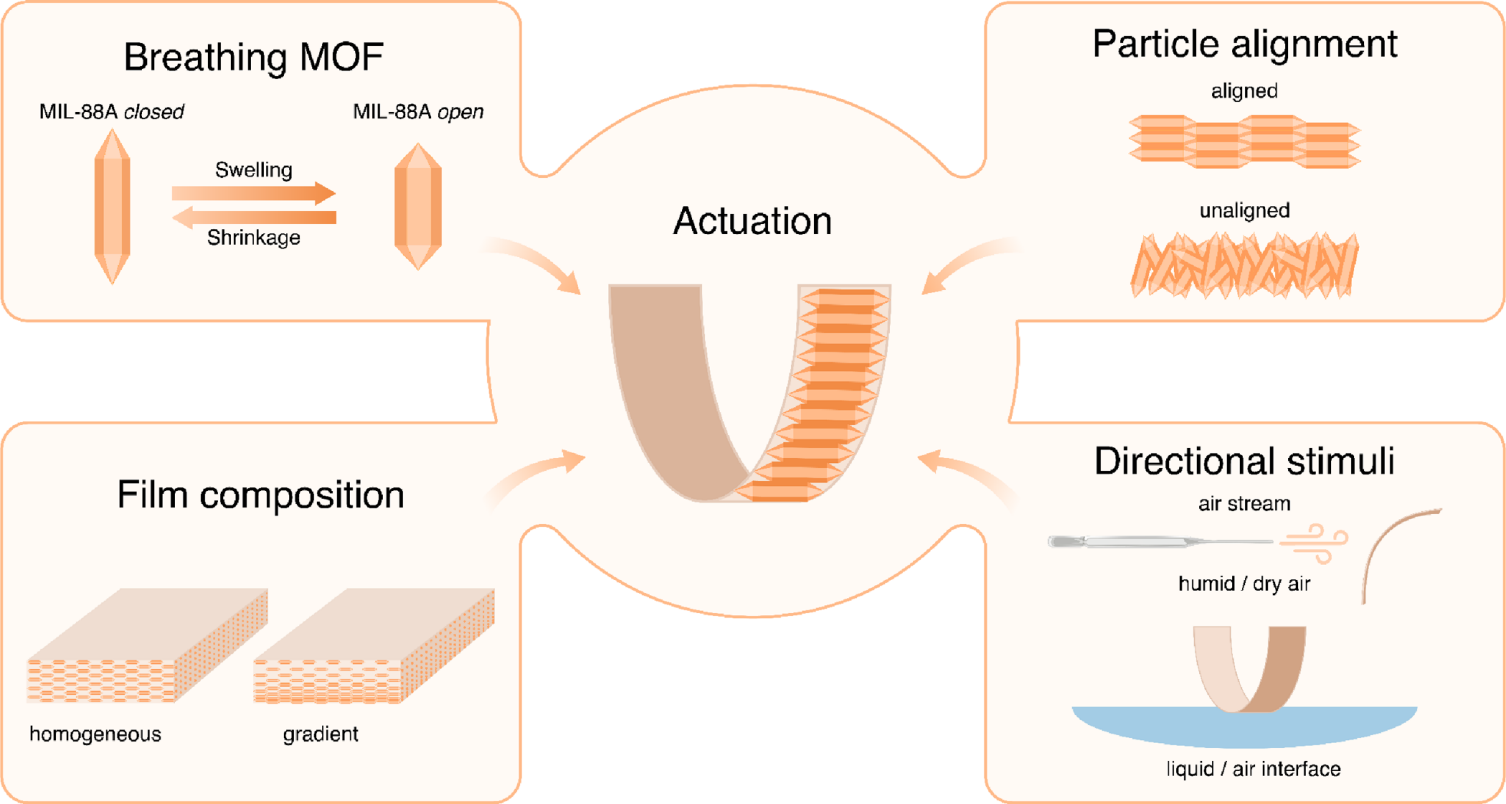
Factors influencing film actuation.

In order to translate the actuation behavior of the MIL-88A into a more practical macroscopic object, we fabricated the MIL-88A@PEGDA polymer films. To harvest the full potential of the developed films, we evaluated how different anisotropies within the film and stimuli direction affected the actuation. Specifically, we studied how the MOF particle alignment, film composition, and directional triggers influenced the film actuation (Figure 6).

We started our experiments with a series of small films (aligned−homogeneous, unaligned–homogeneous, aligned–gradient, and unaligned–gradient). Films with a homogeneous distribution of MOFs were prepared to evaluate the isolated effect of aligned particles. Films with a vertical gradient distribution of MOFs were prepared to afford an active and passive domain to the films in order to enhance the actuation. The aligned-gradient small film shows the highest degree of curvature among the films, owing to the combined effect of the particle’s directional orientation and the vertical gradient generated via sedimentation (SI 2.4, Figure S7). As illustrated in Figure 2, the aligned MIL-88A particles are oriented horizontally along the short side of the film. When the aligned MIL-88A particles in the aligned-gradient small film absorb water, the directional expansion of the MIL-88A propagates along the long axis of the polymer film and the increased expansion of the MOF-rich side results in a higher degree of curving. In the unaligned-gradient small films, the gradient distribution of the particles creates enough anisotropy within the film to observe a folding response; however, the swelling behavior of MIL-88A is not efficiently propagated to the macroscale unless the particles are uniformly oriented. In the small unaligned films, the particles expand in different directions upon water absorption, thus creating a relatively isotropic expansion that does not lead to a degree of folding as large as if the particles are all expanding in the same direction.

**Figure 7:**
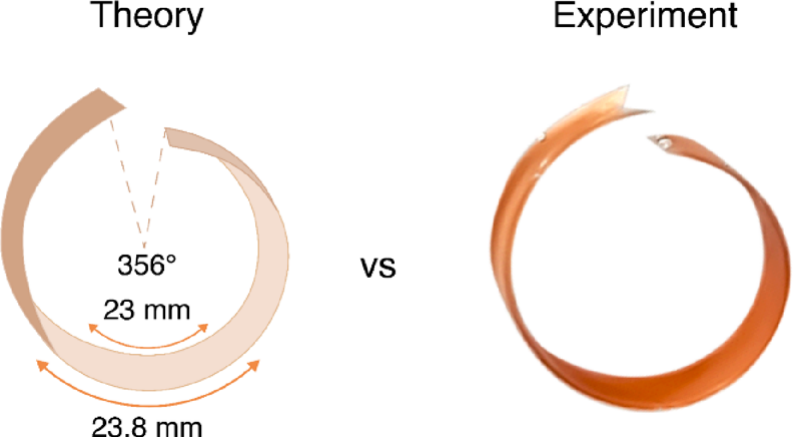
Theoretical calculation of potential folding and a real photo of the folded film.

The theoretical folding of the aligned film can be estimated from geometric considerations based on the volumetric concentration of MOF particles (SI 4). The volumetric concentration estimated from SEM in the top and bottom layers allowed us to calculate the maximum theoretical expansion of each layer, assuming that all rod particles are ideally ordered and transfer their maximum 33% volume expansion to the film. For the small film (aligned-gradient-small), the calculated angle is 356°, which is very close to a full circle of 360° and agrees very well with the experimental observations of the small film immersed in water (Figure 7).

**Figure 8:**
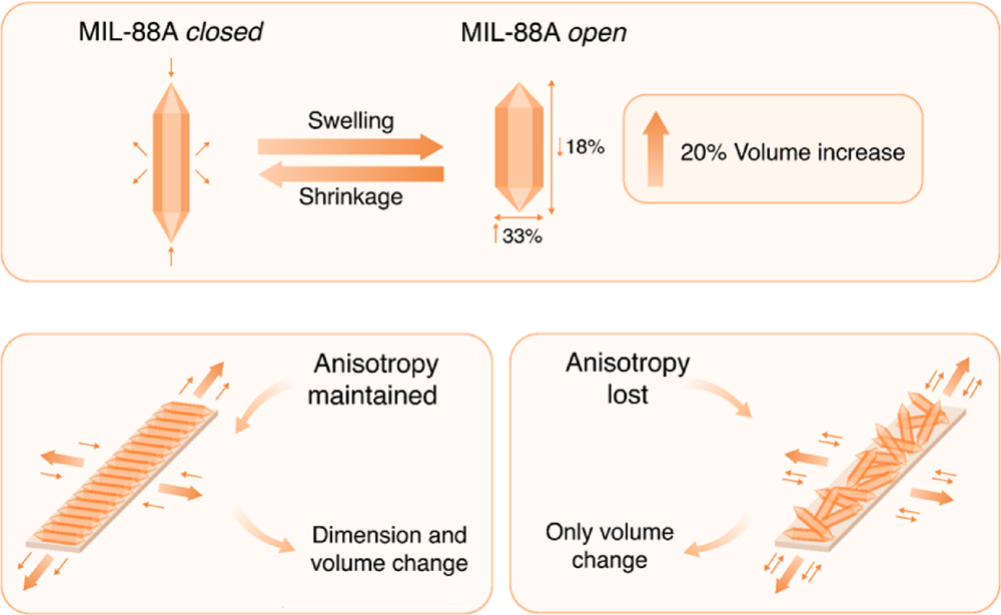
Comparison of the dimension and volume change during swelling and shrinkage of MIL-88A particles in the aligned and unaligned films.

Similar bending behavior was observed in the large film. The aligned-gradient film submerged in water shows a faster and higher degree of curvature compared to the film without alignment (unaligned-gradient). This confirms the propagation of the directional expansion of the individual MIL-88A crystals. When the rod crystals are directionally aligned, the swelling occurs in the same direction throughout the film, increasing the mechanical strain on the passive domain, creating a higher degree of folding. With the unaligned MIL-88A, the MOF particles are swelling and shrinking in multiple directions throughout the film, canceling out the anisotropic behavior, and the expansion is achieved only by the total increase of the volume of particles, creating an isotropic expansion of the area with concentrated MOFs, thus reducing the directional folding of the film (Figure 8).

The degree of curvature in the aligned-gradient-large film also shows a consistent and linear response at different levels of humidity as the directional expansion of the MIL-88A particles results in gradation of swelling behavior that translates to the polymer film (Figure 3, SI 3.2, Figure S17). On the other hand, the unaligned-gradient-large film shows random bending behavior, as the unaligned MIL-88A particles expand in all directions as explained earlier, leading to an inconsistent response to the range of humidity applied.

Upon establishing the transfer mechanism of the anisotropic properties of MIL-88A from the microparticles to the films, we focused on how these highly directional films (aligned-gradient-large and unaligned-gradient-large) would respond to directional stimuli. In order to evaluate the bending behaviors, we devised different experimental setups based on two types of directional stimuli: water and humid air (90% RH). As expected, the movement derived from water was much stronger than the one generated by humid air. However, the curving of the films in water is area-dependent, thus resulting in complex movements and a behavior difficult to quantify. Humid/dry air cycles (SI 3.3, SI Video 5) proved to be much more reliable, as the small nozzle used to direct the stream kept a sufficient constant interaction between the films and the stimuli source (humid/dry air) during movement as well as consistency between different experiments.

For films placed on the water surface, two different movements were observed, depending on the side of the film that was in contact with water (SI 3.1, Table S2, Figure S14). The major movement was observed when side 1 came into contact with the water, which is the curling of the film along its axis in the direction away from the humid air source. This agrees with what was observed so far. The higher degree of curvature and the response speed, compared to that of the films exposed to homogeneous stimuli, is logically attributed to the lack of expansion of the other side of the film. The movement observed when exposing side 2 of the film to the water, namely, curling along the short axis of the film, is more complicated, as its directional movement (toward or away from the water source) is arbitrary for all films. It is most likely due to the very low concentration of the particles on side 2, so the movement of the film is no longer dominated by the swelling behavior of MIL-88A particles, but the combined effects of residual stresses in the polymer film, the swelling effect of the polymer, and the swelling behavior of MIL-88A. The pure polymer film showed the same preferential movement when directionally interfaced with water (SI 3.1, Table S2). This is a direct consequence of the non-uniform swelling of the polymer, while the change in directions is most likely due to the degree of polymerization of the different faces of the film. As the film is photopolymerized with a UV lamp mainly from the top, the lower side has fewer interconnections, a property that strongly influences the swelling of PEGDA polymers.^44,45^ This different degree of interconnection is even stronger for the films containing MOF particles, as MIL-88A absorbs in the UV region of the spectra,^46^ which was corroborated during the fabrication, as addition of MIL-88A required significantly longer UV radiation time to polymerize the films (SI 2.3). The influence of the polymer is only negligible in the case of aligned particles without a gradient in the film. In this case, the film will move along its longer axis independently of which side is put in contact with water as the changes in size and volume guide the bending preferentially along this axis. The unaligned-homogeneous film, on the other hand, follows the polymer’s preferential movement, as the unaligned particles do not have a preferential bending direction.

The directional interaction of humid air and water showed very similar results, with the large aligned-gradient film (aligned-gradient) outperforming both unaligned-gradient and aligned-homogeneous films in movement amplitude. The movement dynamic was the same, with a curling of the film away from the humid air source when the stream of humid air was directed on the MOF-rich side face (side 1) and a minor straightening of the film when it was directed on the polymer-rich side (side 2). Exposure to dry air (<20% RH) triggered an opposite movement of the films, as the MIL-88A pores close, resulting in contraction.

The cycle stability and the strength of the film movement were further investigated. Cyclability was gauged over 20 humid/dry cycles, during which the film performed well, with no substantial change in the overall performance. It is worth noting that the film did not need any activation or drying process throughout the whole duration of the experiment, apart from the drying action of the low-moisture air stream, and that the film movements between resting positions are faster than the one previously reported (seconds instead of minutes), making the film suitable as an actuator in a much wider range of practical conditions.^36^ The improved speed may be due to the high hydrophilicity of PEGDA, which improves the diffusion of water from the external environment, through the polymer to the MOF. The adsorption process is sped up by avoiding the shielding of the particles, while the desorption process is favored as the polymer will bridge the transition of water from the MOF to dry air by a layer with intermediate affinity, low enough to allow water evaporation but high enough to allow diffusion of water molecules from the MOF pores to the polymer.^40^

Measuring the energy involved in the film movement is a complex task due to the minute forces involved. Therefore, we decided to simplify the overall film movement to a purely linear one and rely on a simple but effective design already tested for similar tasks. The dynamometer we designed is based on very reliable and precise mechanical hygrometers. The underlying principle concerns the linear movement of a single strand of hair translated to the rotational movement of an axle, while a calibrated weight or spring keeps the hair under tension. By measuring the angle or rotation of the axle, we were able to directly measure the distance of linear movement of the film, and by using different tensioning weights, we were able to easily change the force applied to the film. Knowing the distance of movement and the tensioning force allows us to easily calculate the energy output of the film, quantifying the mechanical work upon actuation. The large aligned-gradient film is able to move a weight 28 times heavier than itself for a distance of 0.8 mm against gravity (equivalent to a man of 70 kg lifting a 2000 kg weight) when subject to a directional change of humidity from 10 to 90%. Overall, the film showed a linear behavior over a wide range of weights and a constant energy output. When triggered with water by placing droplets on the film surface, the energy output is superior, but it is much more difficult to quantify the data reliably and reproducibly, due to the variation of the film area covered by the water. Our calculations are based on the approximation that the dynamometer suffers no energy loss due to attrition or stretching of the cables, but the constant energy output supports this simplification; if these forces were sufficiently high, the energy output should decrease noticeably with heavier weights.

## Conclusions

In this work, we investigated the effect of harnessing anisotropy at the micro and macro scales on the water-driven actuation of MIL-88A@PEGDA film composites. We demonstrated that the alignment of breathable MOFs propagates their directional expansion to the macroscale, thus increasing directional folding of the films. The use of an E-field to rapidly achieve large areas of particle orientation was showcased as a new tool for fabricating anisotropic MOF materials. Furthermore, we show how the enhanced actuation due to the translation of the MIL-88A expansion results in an increase in mechanical work, as evidenced by the dynamometer measurements. Moreover, by choosing a hydrophilic polymer as the film matrix, we were able to improve movement speed and avoid the need of intervention to reactivate MIL-88A for each adsorption cycle, further improving the applicability of these systems.

This work shows the importance of harnessing anisotropic properties of MOF crystals and the enhanced performance of the material that ensues. Given that most MOFs have anisotropic properties, our results have broad implications for enhancing the performance of MOF composites for applications such as sensing, actuation, separations, and conduction and for optics. The translation of the anisotropic MOF properties to the macroscale object opens the door to bridging the gap between the functionality of ordered lattices and MOF-based composites.

## Experimental Section

### Chemicals

FeCl_3_·6H_2_O (Acros Organics, 99%), fumaric acid (Acros Organics, 99%), Pluronic F127 (Sigma-Aldrich, average Mw = 12,600 g/mol, Aldrich), acetic acid (Thermo Scientific, 99.7%), poly(ethylene glycol) diacrylate (Sigma-Aldrich, average Mn = 250), PEGDA_250_, poly(ethylene glycol) diacrylate (Sigma-Aldrich, average Mn = 700) PEGDA_700_, TMPTA (Sigma Aldrich), and diphenyl(2,4,6-trimethylbenzoyl)phosphine oxide (Sigma-Aldrich, 97%) were purchased and used without further purification.

### Synthesis of MIL-88A

The synthesis of rod-shaped MIL-88A was adapted from literature.^41^ In a typical synthesis, Pluronic acid F-127 (800 mg) was dissolved in Milli-Q water (66.7 mL) by stirring at 600 rpm for 10 min. 16.6 mL of a 0.4 M FeCl_3_·6H_2_O aqueous solution was added, and the mixture was stirred for 1 h at 600 rpm. Acetic acid (1.5 mL) was added, and the solution was left to stir for 1 h at 600 rpm, followed by addition of fumaric acid (780 mg). The orange suspension was left to stir for 2 h at 600 rpm and then transferred to two Teflon-lined autoclaves and heated to 110 °C for 24 h. Upon cooling to room temperature, the product was recovered via centrifugation (10,000 rpm, 10 min). MIL-88a was washed three times with EtOH (10,000, 10 min) and redispersed in EtOH.

### MIL-88A Alignment in the Electric Field

A solution of MIL-88A in various solvents was prepared (EtOH, DMA, DMF) and placed in a sonicator to disperse the MOFs. A 0.5 mm × 10 mm capillary was used to take out an aliquot, and the two ends were sealed with a flame gun. The capillary was placed flat in between the two electrodes on the glass slide described previously, and the two silver wires were connected to a wave-function generator coupled to an amplifier to apply the E-field. The sample was observed under the microscope to see if the MOF particles oriented and chained in the direction of the E-field. The high dielectric constant of the solvent allowed for screening of MIL-88A intermolecular forces, and thus chaining and packing were observed during the alignment.^20^ Upon turning off the E-field, the MIL-88A rods immediately returned to a random orientation.

### Fabrication of MIL-88A@PEGDA Films

Small films (dimension: ca. 2 × 0.4 cm): A 9:9:1 ratio of polymer solution composed of PEGDA_250_ and PEGDA_700_ and TMPTA was prepared. Dried MIL-88A (4 mg) was dispersed in the polymer solution (16 μL) using a sonicator for 5 min. 4 μL of TPO stock solution made from 4 mg of TPO in 40 μL of DMA was added to the MOF polymer dispersion, and it was sonicated for another minute. The MIL-88A dispersion was then added to the customized channels made by cover slide glasses with spacers of thickness 0.12 mm. A wave-function generator coupled to an amplifier was then used to apply an E-field (100 V/mm). Homogeneous and gradient distributions of MIL-88A particles in polymer films were fabricated in 15 min and 1 h, respectively. With the E-field still on, a SUNmini2 UV lamp (6 W, 365 nm + 405 nm) was used to cure the polymer matrix. Once the films were polymerized, they were removed from the channel slide and soaked in EtOH for 10 min before being left to dry under air at room temperature. For the unaligned films, the same conditions were applied without the E-field.

Large films (dimension: ca. 5 × 38 × 0.2 mm): A 9:9:1 ratio of polymer solution composed of PEGDA_250_ and PEGDA_700_ and TMPTA was prepared. Dried MIL-88A (16 mg) was dispersed in the polymer dispersion (64 μL) using a sonicator for 10 min. TPO (2.4 mg) and DMA (24 μL) were added to the MOF polymer solution, and it was sonicated for 5 min. A 0.2 mm-deep sticky channel slide was sealed with fluorinated ethylene propylene (FEP) film. The slide was placed with the FEP side down on a customized slide containing the electrodes (S1.2, Figure S1) and connected to a wave-function generator coupled to an amplifier to apply the electric field (100 V/mm). With the electric field on, the MOF polymer solution (80 μL) was loaded through the Luer ports to have a homogeneous distribution of the solution. The mixture was left in the dark and under the E-field for 1 h or 15 min to fabricate gradient or homogeneous films, respectively. With the E-field still on, a UV lamp with 6 W power was used to preliminarily polymerize the polymer matrix for 10 min, followed by using a stronger in-house built UV lamp (100 W, 365 nm) for 3 min to fully polymerize the films. Once the films were polymerized, they were removed from the channel slide and soaked in EtOH for 30 min before being left to dry under air at room temperature. For the unaligned samples, the channel slides were placed on top of a glass slide instead of the electrode.

The solo polymer film was fabricated using the same oligomer ratios and TPO; only the polymerization is completed using only the 6 W UV lamp.

### Film Energy Output Measurements

The movement of the film was measured in degrees (presented data are the average of three measurements). For each weight, the film was allowed to relax at ambient humidity and the instrument was zeroed. The film was then subjected to a stream of dry air until stable, and the position was recorded. Then, the stream was switched to humid air and the instrument was again allowed to reach stability and the position recorded. Tweezers were gently positioned against the film to stabilize it during exposure to the air flow and retracted in order to read the movement. The angular movement was then transposed to a linear motion with the conversion factor obtained previously (1° = 0.0178 mm), and the work was calculated following the standard formula of Newtonian physics.

## Supporting Information Available

Instruments used, full description of material synthesis, complete characterizations, comprehensive film movement studies, theoretical estimate, film energy output (PDF)
Supporting video 1: Aligned gradient film behavior to water stimuli (MP4)
Supporting video 2: Unaligned gradient film behavior to water stimuli (MP4)
Supporting video 3: Aligned homogeneous film behavior to water stimuli (MP4)
Supporting video 4: Unaligned homogeneous film behavior to water stimuli (MP4)
Supporting video 5: Aligned gradient film cyclability to humid/dry air flow (MP4)
Supporting video 6: Aligned gradient film dynamometer measurement (MP4)
