# Supplementary material for: Alignment of Breathing Metal–Organic Framework Particles for Enhanced Water-Driven Actuation
Source: Chem Mater. 2023 Aug 25;35(17):6943–52. doi: 10.1021/acs.chemmater.3c01186 (PMC10500993; doi:10.1021/acs.chemmater.3c01186)
Supplement: Supplementary file 1 — cm3c01186_si_001.pdf [file cm3c01186_si_001.pdf]

Supporting Information

Alignment of Breathing Metal-Organic Framework Particles for Enhanced Water-Driven Actuation

Jacopo Andreo,[a,†] Alejandra Durán Balsa,[b,†] Min Ying Tsang,[b] Anna Sinelshchikova,[a] Orysia Zaremba,[a] Stefan Wuttke,[a,c,*] and Jia Min Chin[b,*]

[a] BCMaterials, Basque Center for Materials, Applications and Nanostructures, UPV/EHU Science Park, Leioa 48940, Spain

[b] Faculty of Chemistry, Institute of Inorganic Chemistry - Functional Materials, University of Vienna, Währingerstr. 42, Vienna A-1090, Austria

[c] Ikerbasque, Basque Foundation for Science, 48009, Bilbao, Spain

†These authors contributed equally to this work.

Corresponding authors: stefan.wuttke@bcmaterials.net, jiamin.chin@univie.ac.at

Table of contents

**SI 1**	**Instrumentation**	**3**
1.1	Optical microscopy	3
1.2	Electric field generator and electrodes	3
1.3	Powder x-ray diffraction (PXRD)	4
1.4	Scanning electron microscopy (SEM)	4
1.5	Fourier Transform Infrared Spectroscopy (FTIR)	4
1.6	Thermogravimetric analysis (TGA)	5
1.7	Humidity Chamber	5
1.8	Custom dynamometer fabrication	5
1.9	Dynamometer calibration	6
1.10	Humid/dry air flow setup	7
**SI 2**	**Synthesis and Characterization**	**9**
2.1	Chemicals	9
2.2	Synthesis of MIL-88A	9
2.3	MIL-88A alignment in electric field	9
2.4	Fabrication of MIL-88A@PEGDA films	11
2.5	Characterization of MIL-88A@PEGDA films	14
2.5.1	Optical microscope images	14
2.5.2	PXRD patterns	15
2.5.3	SEM images	16
2.5.4	FTIR spectra	17
2.5.5	TGA analysis	19
**SI 3**	**Film movements investigation**	**20**
3.1	Film movement in water	20
3.2	Film response to the change of humidity	24

3.3 Directional movement of films with air stream 28

SI 4 Theoretical estimate 29

SI 5 Film energy output 31

SI 6 References 32

SI 1 Materials and Instruments

1.1 Optical microscopy

Microscope images were captured on a Zeiss Axio Imager.M2m microscope connected to a Zeiss 208 color Axiocam, and processed using ZEN microscopy software.

1.2 Electric field generator and electrodes

The AC E-field was applied using a wave function generator (Siglent SDG 1032X Function/Arbitrary Waveform Generator) coupled to an amplifier (KROHN-HITE Wideband Power Amplifier 7600). Electrodes were prepared on a glass slide (75 mm x 25 mm) in the following manner: two pieces of double-sided carbon tape were placed along the length of the slide 7 mm apart. Two silver wires were placed on the carbon tapes and covered with copper tape, leaving free wire on each side to serve as electrical contacts. Using two spacers with height of 0.12 mm, a thin coverslip was placed between the electrodes, creating a smooth surface to place the channel slide. The silver wires from the electrodes were connected to the amplifier using electrical wires with crocodile clamps.

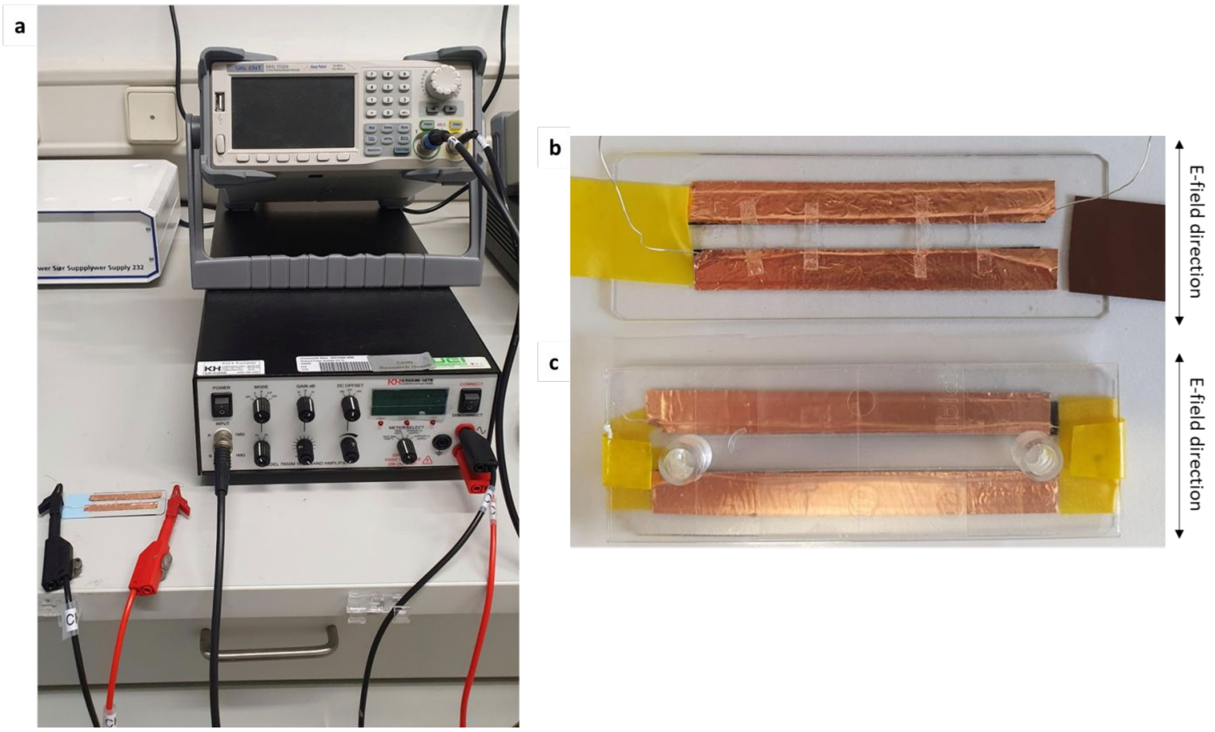

Figure S1. a) Wave function generator and amplifier, Electrode setup used for aligned b) small film and c) large film fabrication.

1.3 Powder X-ray diffraction (PXRD)

Powder X-ray diffraction (PXRD) measurements were carried out on an Empyrean Panalytical in Reflection-transmission spinner configuration to confirm the identity of the synthesized MOFs and to confirm their structural retention after MOF/polymer composite preparation. The Anode Material is Cu, Step Size [˚2θ] is 0.0130, Generator Settings were 40 mA, 45 kV, Measurement Temperature [˚C] was 25.

1.4 Scanning electron microscopy (SEM)

Scanning electron microscope (SEM) images of the MOF particles and MOF/polymer film composites were captured on a Hitachi TM4000 plus electron microscope at 10 kV.

1.5 Fourier Transform Infrared Spectroscopy (FTIR)

Fourier Transform Infrared (FTIR) spectroscopy was carried out in a Bruker TENSOR 37.

1.6 Thermogravimetric analysis (TGA)

Thermogravimetric analysis (TGA) was done on a NETZSCH STA 449 F3 Jupiter under air (from a gas bottle, mix = 80/20). Samples were placed to lie flat on the bottom of Al_2O_3 crucibles and heated from room temperature to 800 ˚C at 10 ˚C/min.

1.7 Humidity Chamber

Controlled relative humidity (RH) experiments were done in a BINDER MKFT 56 Alternating climate chamber at 25 ˚C, testing RH values from 20% to 98%.

1.8 Custom dynamometer fabrication

The dynamometer is composed of a brass frame (Figure S2a, bars with section of 20 x 2 mm connected by bolts to angular 10 x10 mm brackets, 1 mm thick), an axle of stainless steel (Figure S2b, diameter 1.98 mm) is held captive between the two lower crossbars (Figure S2c). Its extremities, sharpened to a 60° point, rest in two punches hammered in the brass with a 50° taper. This geometry ensures the

smooth rotation of the axle minimizing surface contact and thus attrition. Mounted on the axle is a double-sided hand and nine spires of braided 0.06 mm PE fishline. The fishline ends with an open ring on one side and holds a weight on the other. For weight, different combinations of calibrated bolts and nuts were used (Figure S2d). A dial with 360 divisions is mounted on the rear crossbar, centered on the axle. The top bar (Figure S2e) holds captive a M3 screw used for fine adjustment of the instrument, with a jam nut to block it securely during measurement and a swivel joint on the tip, in order to ensure easy positioning of the film. The film (Figure S2f) is held in tension between the fixed swivel hook and the open ring fixed to the axle by two lengths of fish line with rings on the ends glued at the ends of the film.

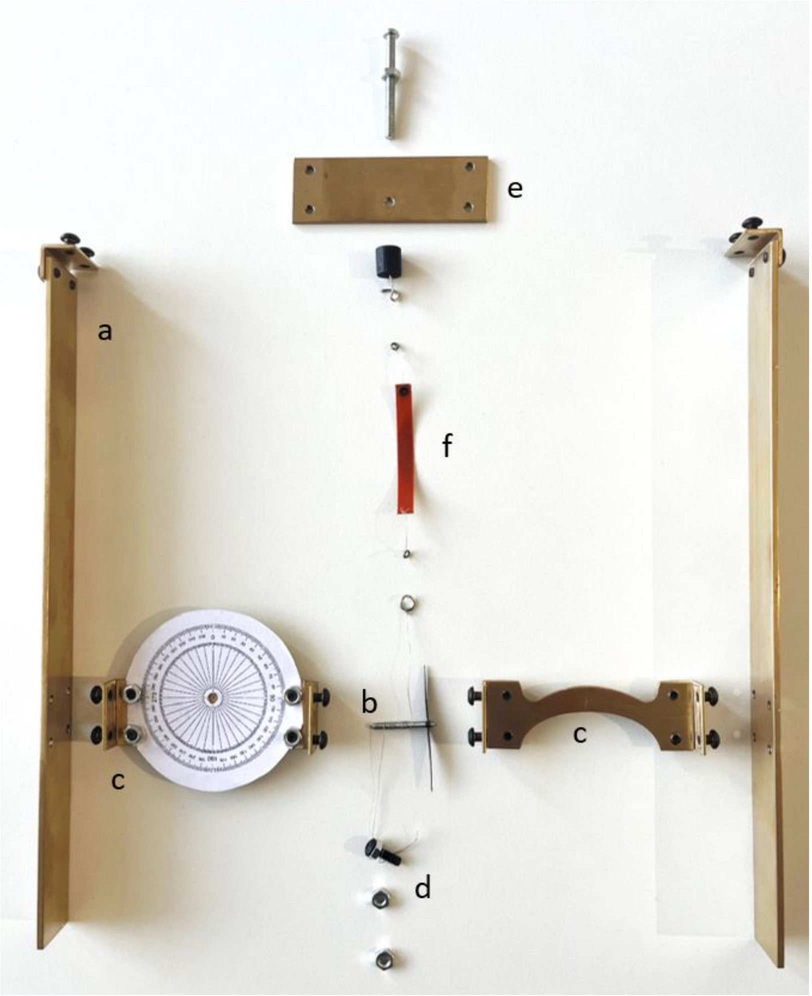

Figure S2. Dynamometer layout.

1.9 Dynamometer calibration

The relation between linear and angular movement of the dynamometer was directly measured on the instrument, by switching the flexible film for a length of fish line, zeroing the instrument and then measuring the distance of the adjusting screw to the top crossbar after moving the instrument hand of a fixed number of degrees (Table S1, values mediated on 3 measurements). The collected measurements showed good linear fitting and led to a value of 6.41 mm of travel for rotation, value in

agreement with the one calculated by measuring the axle diameter, adding the thickness of the line and calculating the circumference (6.41 mm).

Table S1. Calibration curve of the relation between degrees of movement and distance of movement

Dynamometer calibration											
degrees	-180	-160	-120	-80	-40	0	40	80	120	160	180
mm	3.03	2.63	2.12	1.33	0.66	0	-0.66	-1.37	-2.25	-3.08	-3.42

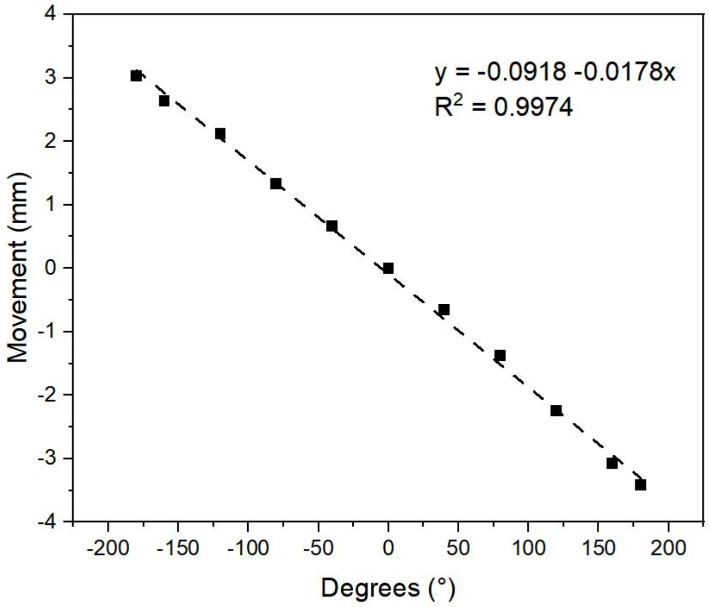

Figure S3. Calibration curve of the relation between degrees of movement and distance of movement.

1.10 humid/dry air flow setup

The humid/dry air flow was obtained using an aquarium pump with two outlets (each with a flow of 5 l/m, Figure S4a). The two lines were connected in line to a filter and a gas washing bottle via silicon tubing. The gas washing bottles were filled respectively with water (Figure S4b) or drying agent (Silica gel LABKEM with orange indicator, Figure S4c). The output nozzle (a Pasteur pipette with a tip diameter of 0.7 mm, Figure S4d) was manually connected to the required bottle. The streamed air had a humidity over 90% for the water filled bottle and lower than 20% for the silica gel one.

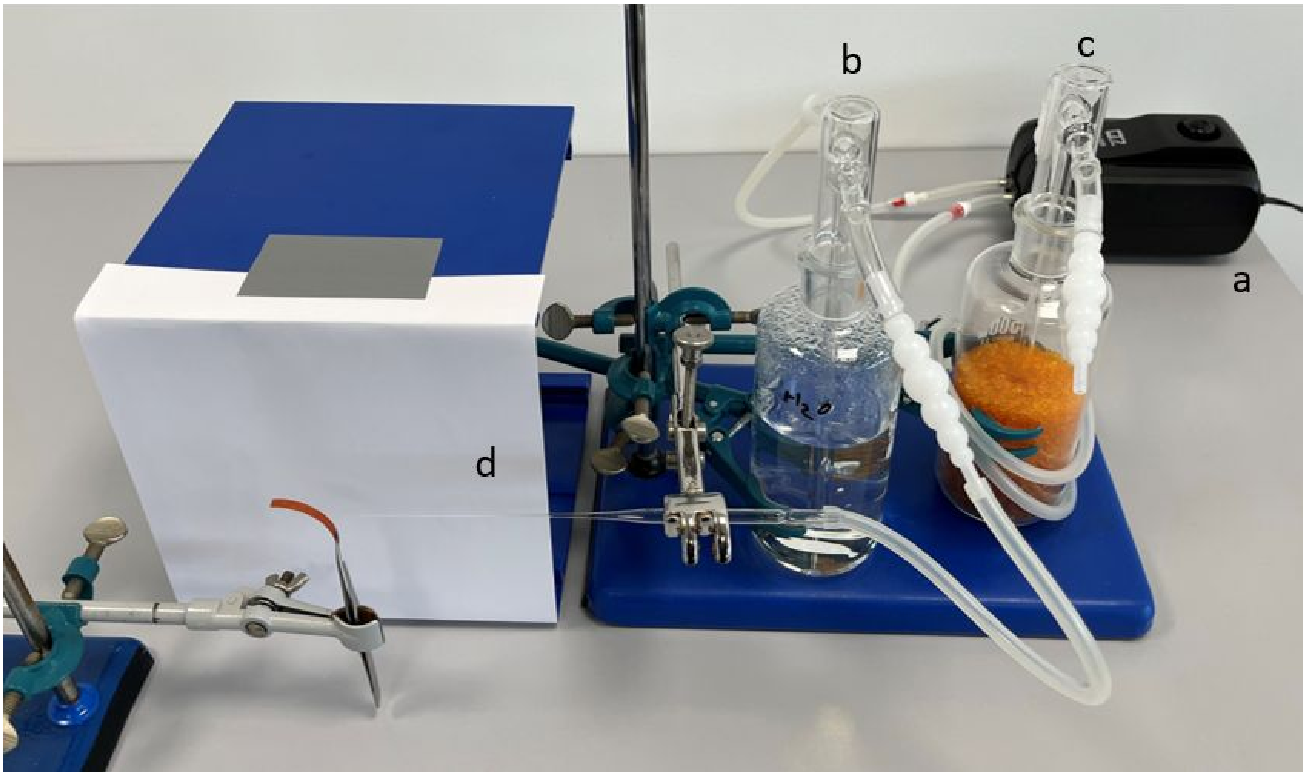

Figure S4. Detailed setup of air flow directed perpendicularly to the film through a small nozzle

SI 2 Synthesis and characterization

2.1 Chemicals

$FeCl_3 \cdot 6H_2O$ (Acros Organics, 99%), fumaric acid (Acros Organics, 99%), Pluronic F127 (Sigma Aldrich, average Mw = 12 600 g/mol, Aldrich), acetic acid (thermo Scientific, 99.7%), Poly(ethylene glycol) diacrylate (Sigma Aldrich, average Mn = 250) $PEGDA_{250}$, Poly(ethylene glycol) diacrylate (Sigma Aldrich, average Mn = 700) $PEGDA_{700}$, Trimethylolpropane triacrylate (Sigma Aldrich), Diphenyl(2,4,6-trimethylbenzoyl)phosphine oxide (Sigma Aldrich, 97%) were purchased and used without further purification.

2.2 Synthesis of MIL-88A

The synthesis of rod-shaped MIL-88A was adapted from literature.[1] In a typical synthesis, Pluronic acid F-127 (800 mg) was dissolved in Milli Q water (66.7 mL) by stirring at 600 rpm for 10 minutes. 16.6 mL of a 0.4 M $FeCl_3 \cdot 6H_2O$ aqueous solution was added and the mixture was stirred for 1 hr at 600 rpm. Acetic acid (1.5 mL) was added and the solution was left to stir for 1 hr at 600 rpm, followed by the addition of fumaric acid (780 mg). The orange suspension was left to stir for 2 hrs at 600 rpm, then transferred to two Teflon lined autoclaves and heated to 110 ˚C for 24 hrs. Upon cooling to room temperature, the product was recovered via centrifugation (10 000 rpm, 10 minutes). The MIL-88a was washed 3 times with EtOH (10 000, 10 minutes) and redispersed in EtOH.

2.3 MIL-88A alignment in electric field

A solution of MIL-88A in various solvents was prepared (EtOH, DMA, DMF) and placed in the sonicator to disperse the MOFs. A 0.5 mm x 10 mm capillary was used to take out an aliquot and the two ends were sealed with a flame gun. The capillary was placed flat in between the two electrodes on the glass slide described previously and the two silver wires were connected to the wave function generator coupled to an amplifier to apply the E-field. The sample was observed under the microscope to see if the MOF particles oriented and chained in the direction of the E-field. The high dielectric constant of the solvent allowed for screening of MIL-88A intermolecular forces, and thus chaining and packing was observed during the alignment.[2] Upon turning off the E-field, the MIL-88A rods immediately return to a random orientation. At high frequencies, the dominant mechanism driving the particle orientation is the dielectric polarization of the particle itself.[3] The diacrylate moieties in PEGDA oligomer mixtures afford its polarity, allowing rapid alignment of the well dispersed MIL-88A particles prior to sedimentation. It was observed that the alignment in the polymer solution was slower than in pure DMA and can be attributed to the higher viscosity.

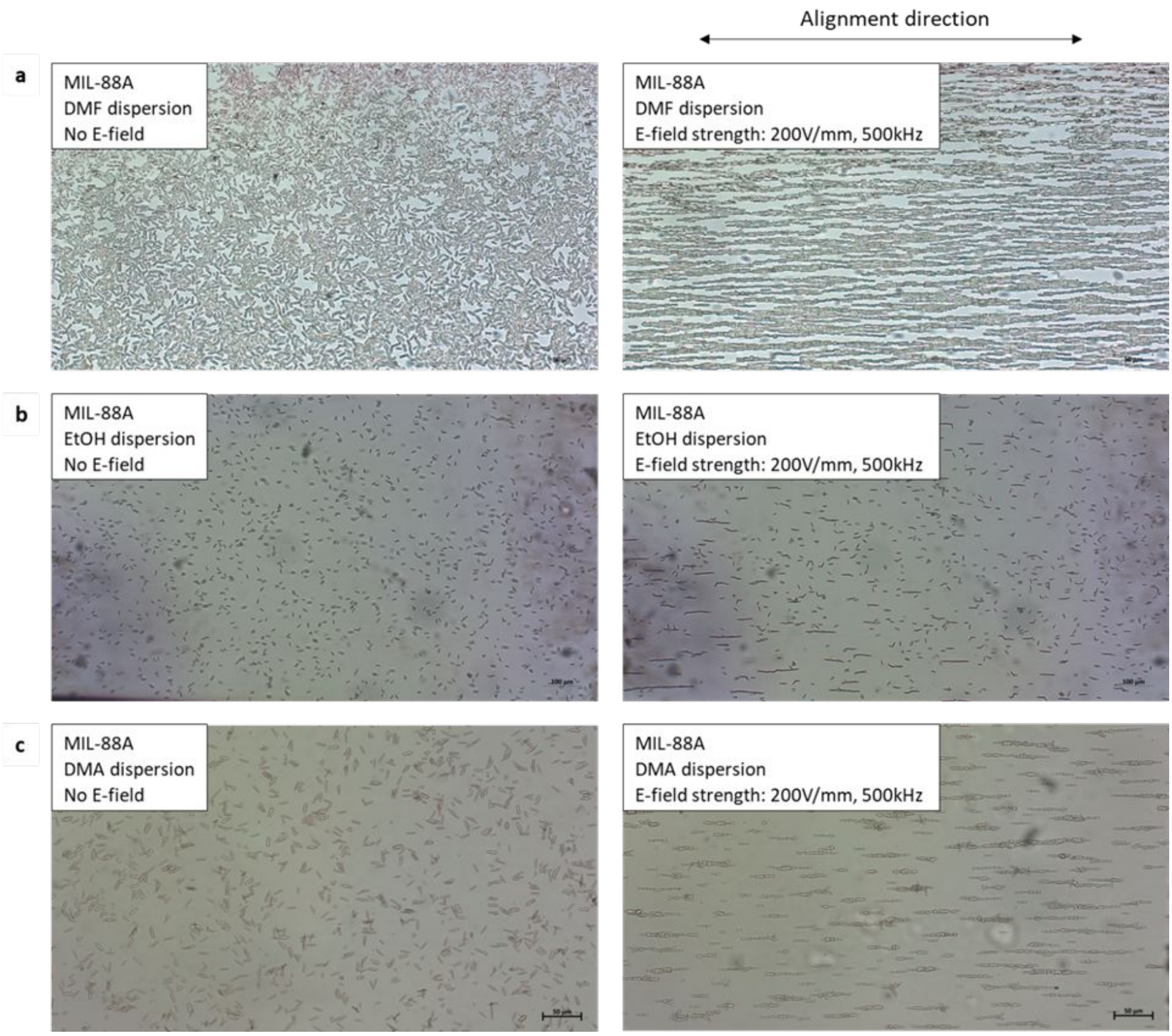

Figure S5. Alignment condition screening under E-field 200 V/mm and 500 kHz: a) in DMF solvent; b) in EtOH solvent and c) in DMA solvent.

2.4 Fabrication of MIL-88A@PEGDA films

Small films (dimension: ca. 2 x 0.4 cm): A 9:9:1 ratio of polymer solution composed of $PEGDA_{250}$ and $PEGDA_{700}$ and TMPTA was prepared. Dried MIL-88A (4 mg) was dispersed in the polymer solution (16 µL) using a sonicator for 5 minutes. 4 µL TPO stock solution made from 4 mg TPO in

40uL DMA was added to the MOF polymer dispersion and it was sonicated for another minute. The MIL-88A dispersion was then added to the customised channels made by cover slide glasses with spacers of thickness 0.12 mm. A wave function generator coupled to an amplifier was then used to apply an E-field (100 V/mm). Homogeneous and gradient distribution of MIL-88A particles in polymer films were fabricated in 15 mins and 1 h, respectively. With the E-field still on, a SUNmini2 UV lamp (6 W, 365 nm + 405 nm) was used to cure the polymer matrix. Once the films were polymerized, they were removed from the channel slide and soaked in EtOH for 10 minutes before being left to dry under air at room temperature. For the unaligned films, the same conditions were applied without the E-field.

Large films (dimension: ca. 5 x 38 x 0.2 mm): A 9:9:1 ratio of polymer solution composed by $PEGDA_{250}$ and $PEGDA_{700}$ and TMPTA was prepared. Dried MIL-88A (16 mg) was dispersed in the polymer dispersion (64 µL) using a sonicator for 10 minutes. TPO (2.4 mg) and DMA (24 µL) were added to the MOF polymer solution and it was sonicated for 5 minutes. A 0.2 mm deep sticky channel slide was sealed with fluorinated ethylene propylene (FEP) film. The slide was placed with the FEP side down on a customised slide containing the electrodes and connected to a wave function generator coupled to an amplifier to apply the electric field (100 V/mm). With the electric field on, the MOF polymer solution (80 µL) was loaded through the Luer ports to have a homogeneous distribution of the

solution. The mixture was left in the dark and under E-field for 1 hour or 15 minutes to fabricate gradient or homogeneous films respectively. With the E-field still on, a UV lamp with 6 W power was used to preliminarily polymerize the polymer matrix for 10 minutes, followed by using a stronger in-house built UV lamp (100 W, 365 nm) for 3 mins to fully polymerize the films. Once the films were polymerized, they were removed from the channel slide and soaked in EtOH for 30 minutes before being left to dry under air at room temperature. For the unaligned samples, the channel slides were placed on top of a glass slide instead of the electrode.

The solo polymer film was fabricated using the same oligomer ratios and TPO, only the polymerization is completed using only the 6 W UV lamp.

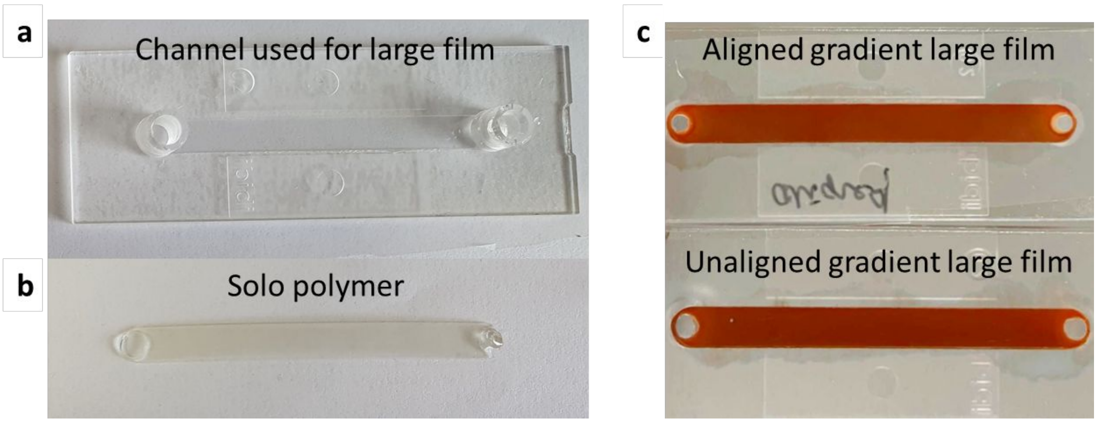

Figure S6. a) Channel used for the large film fabrication; b) solo polymer film; c) aligned (top) and unaligned (bottom) films after polymerization in the channels. The prepared films have an opaque side on the bottom where the MOFs have sedimented and accumulated (side 1) while the top of the film is

glossy (side 2), as there is a layer of mostly PEGDA. The aligned film looks lighter in color than the unaligned film, which can be attributed to less obstruction of light that can pass through the ordered layer of MOFs versus the unoriented MOF layer in the unaligned film.

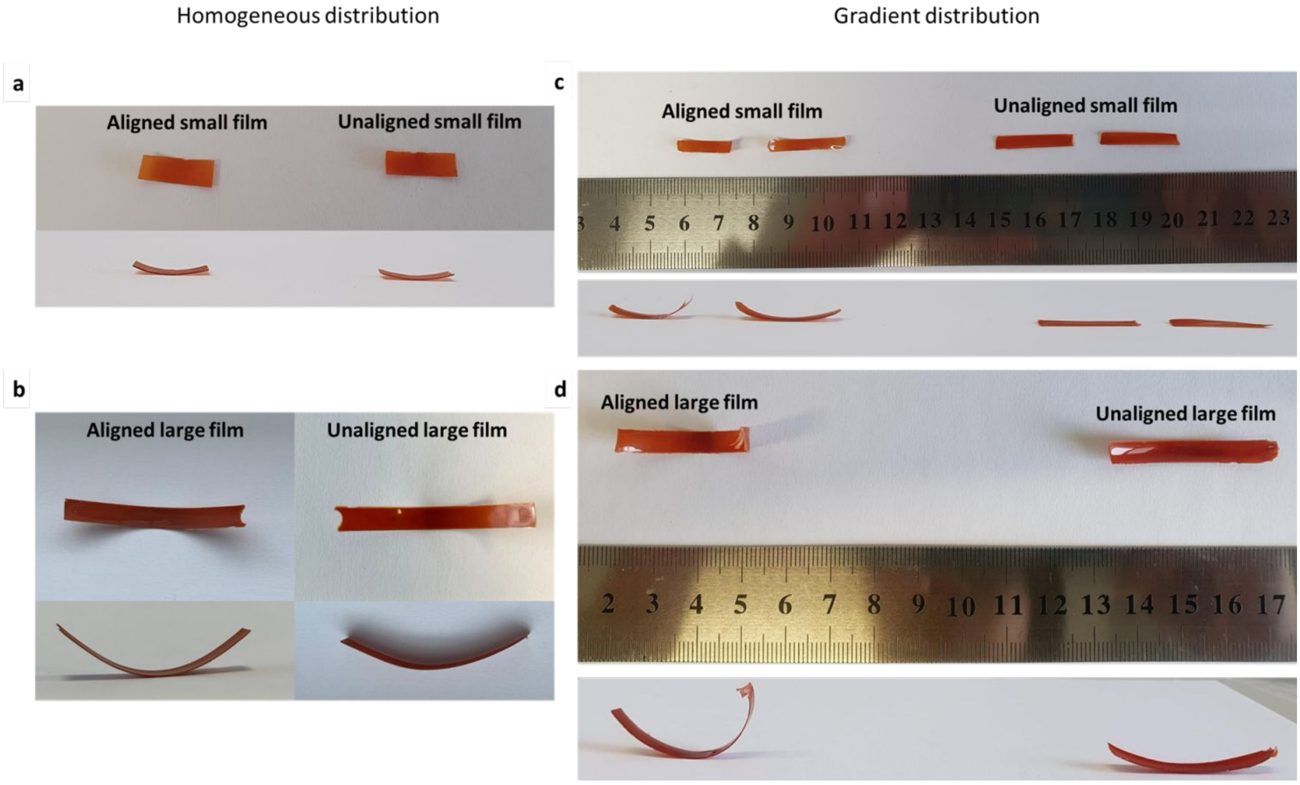

Figure S7. a) small films and b) large films with homogeneous distribution immediately after fabrication and c) small films and d) large films with gradient distribution immediately after fabrication.

2.5 Characterization of MIL-88A@PEGDA films

2.5.1 Optical Microscope images

MIL-88A crystal size (dried versus wet)

The microscopic images show the MIL-88A crystal has size ca. 7.0 x 1.3 µm in dried condition, 6.2 x 1.7 µm in water. The change in height is about - 14% and the change in width is about 32%, concordant with the reported swelling behavior of MIL-88A.[4]

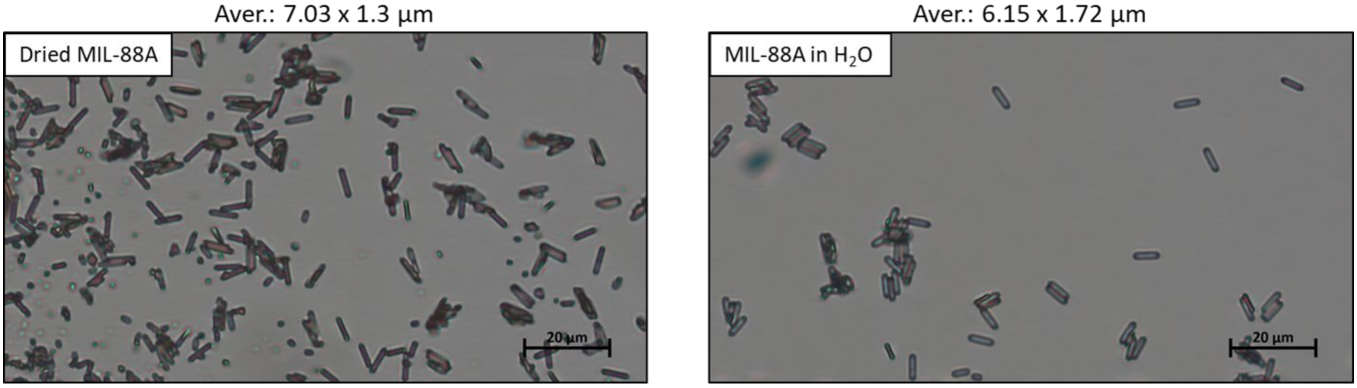

Figure S8. microscopic images of dried MIL-88A (left) and wet MIL-88A in water (right).

2.5.2 PXRD patterns

The synthesized MIL-88A particles were confirmed with PXRD (Figure. S9) and corresponded with the literature.[5] The corresponding 2θ peaks for MIL-88A at 10.4˚ and 12˚ were present in PXRD measurements of the aligned and unaligned MIL-88A@PEGDA films, indicating the structure of MIL-88A is retained during the film fabrication process with and without E-field. The broad peak at about 22˚ corresponds to the PEGDA film due to the amorphous feature of the polymer. All films and dried MIL-88A correspond to the closed form of MIL-88A and the MIL-88A in H_2O corresponds to the open form of MIL-88A, confirmed by comparison with the simulated patterns.

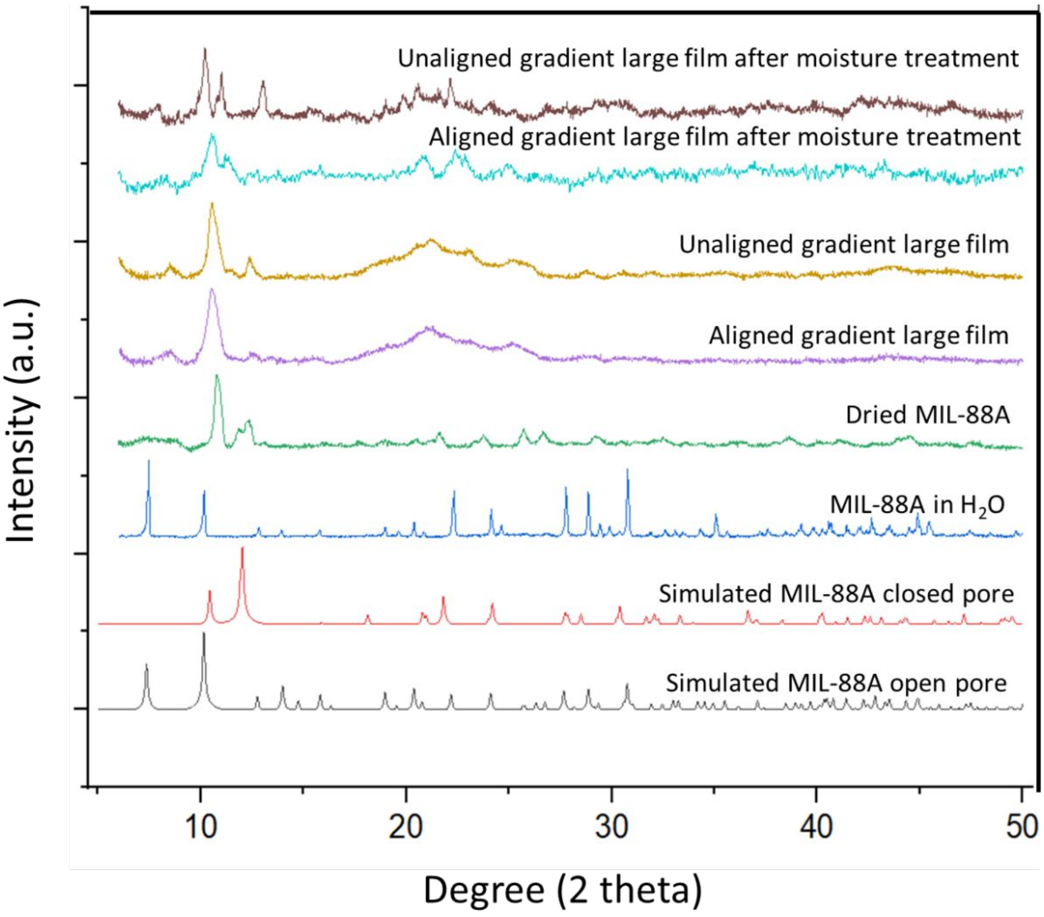

Figure S9. PXRD pattern of MIL-88A in water, dried MIL-88A and the aligned and unaligned MIL-88A@PEGDA polymer before and after moisture treatment. The PXRD patterns of the films present broader bands that are compatible with states between the full open and full closed framework, as reported by Serre et al.[5]

2.5.3 SEM images

A series of small and large films (aligned homogeneous, unaligned homogeneous, aligned gradient and unaligned gradient) were imaged and the aligned particles in both homogenous and gradient versions of aligned films are clearly illustrated (Figure S10 and S11).

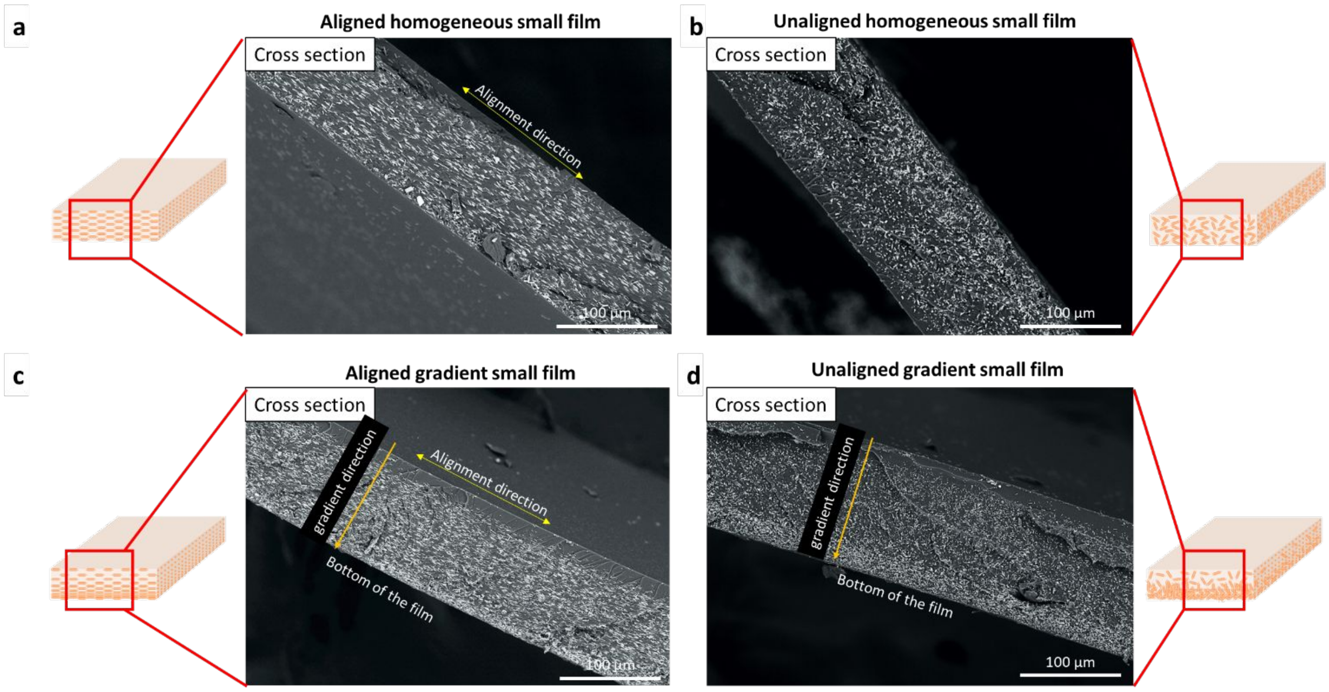

Figure S10. SEM images of a series of small films in different conditions with magnification 300x: a) homogeneous, aligned; b) homogeneous, unaligned; c) gradient, aligned; d) gradient, unaligned.

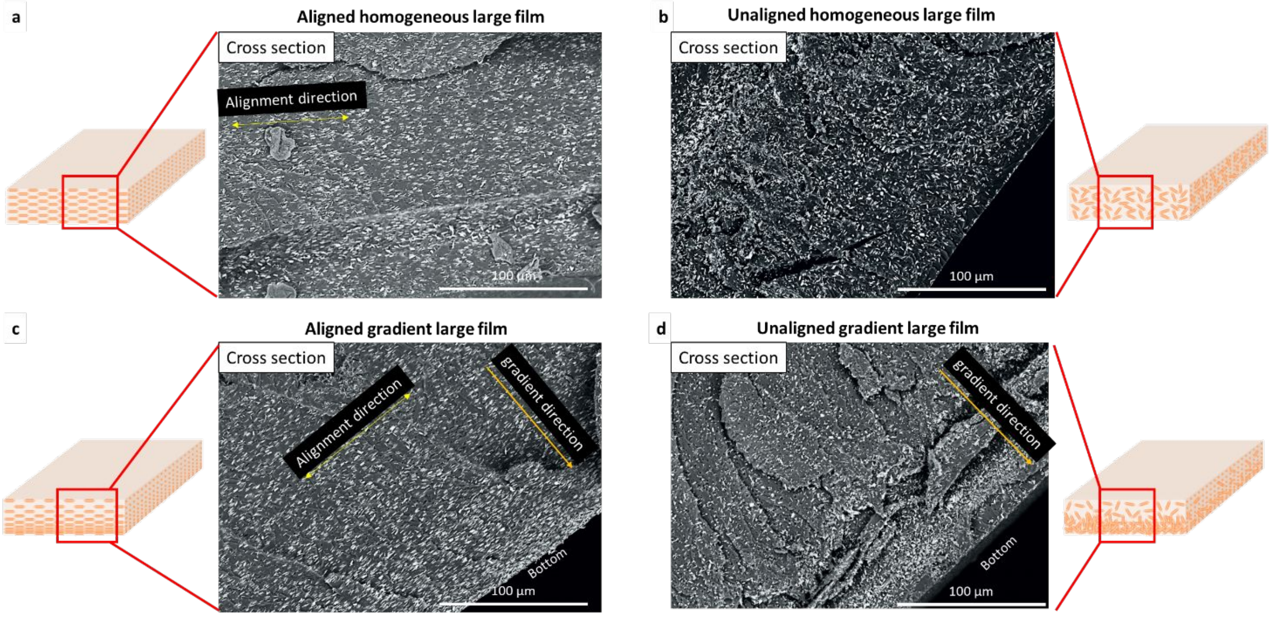

Figure S11. SEM images of a series of large films in different conditions with magnification 500x: a) homogeneous, aligned; b) homogeneous, unaligned; c) gradient, aligned; d) gradient, unaligned.

2.5.4 FTIR spectra

Infrared spectroscopy of the synthesized MIL-88A and the composite films shows expected peaks for MIL-88A (Figure S12). The peaks at 1603 cm^{-1} and 1394 cm^{-1} correspond to the symmetric and asymmetric vibrational frequencies of the carboxyl group from the fumaric acid linker,[6] and the Fe-O vibration is attributed to the band absorption at 566 cm^{-1}.[7] These identifying peaks are present in the IR spectrum of the MIL-88A@PEGDA aligned and unaligned films, further confirming the integrity of the MIL-88A particles after film fabrication.

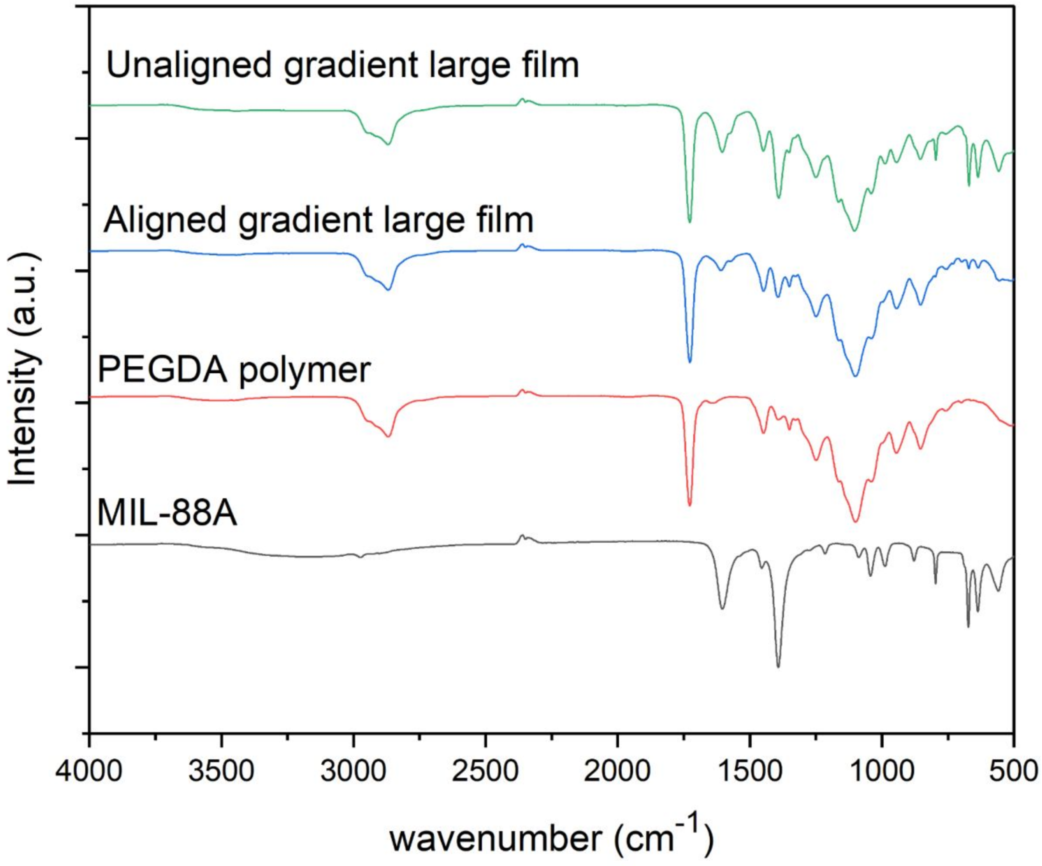

Figure S12. IR spectra of MIL-88A, PEGDA polymer and the aligned and unaligned MIL-88A@PEGDA polymer.

2.5.5 TGA analysis

Thermogravimetric analysis of a vacuum activated MIL-88A@PEGDA film, powder MIL-88A, and PEGDA film was done to estimate the MOF wt% loading in our samples (Figure S12). The TGA curve for powder MIL-88A shows the early mass loss stages between 200 and 300 ˚C corresponding to the fumaric acid and total decomposition at 500 ˚C concurrent with the literature.[8] The residual mass after decomposition was 35.9%. The PEGDA film shows thermal stability until around 300 ˚C and is fully decomposed at 600 ˚C with a 2.3% residual mass. The MIL-88A@PEGDA film showed full decomposition by 550 ˚C with a residual mass of 8.5%. Based on the thermogravimetric curves and residual masses, the experimentally determined MOF loading of the films was calculated to be 17.3%, in agreement with the film fabrication ratio.

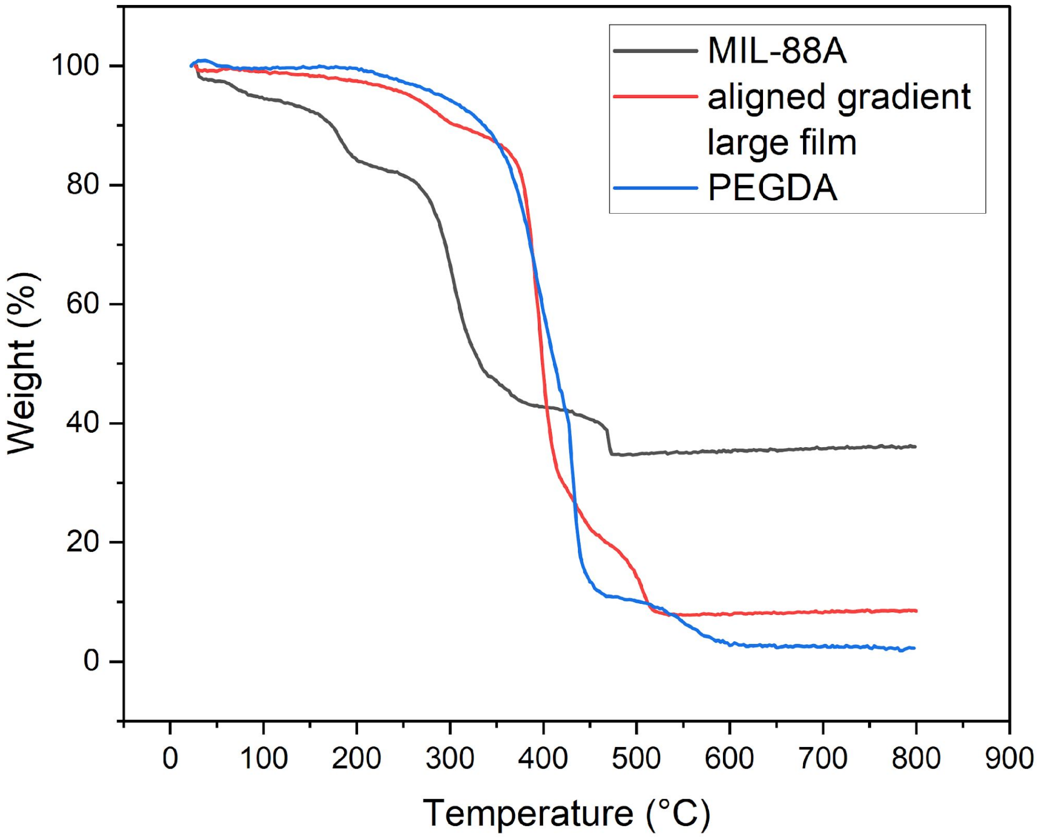

Figure S13. TGA of MIL-88A, MIL-88A@PEGDA and PEGDA polymer.

SI 3 Film movements investigation

3.1 Film movement in water

Table S2. Illustration of the response of MIL-88A@PEGDA large films to static (submerged in water) or directional stimuli (films placed on water surface by different sides).

Film	Static stimuli (submerged in water)	Directional stimuli, side1 (water surface)	Directional stimuli, side2 (water surface)
Aligned gradient film	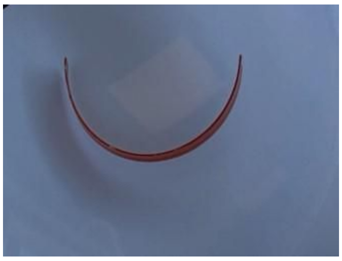	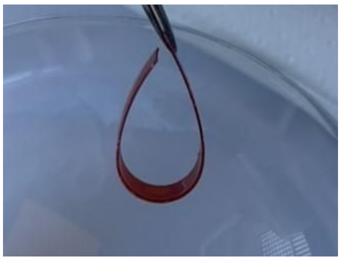	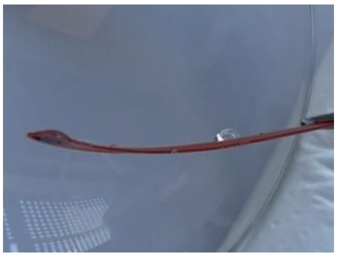
Unaligned gradient film	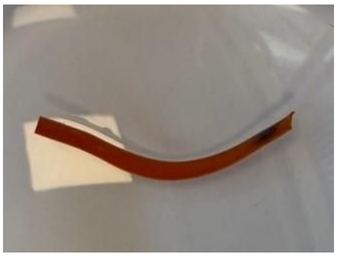	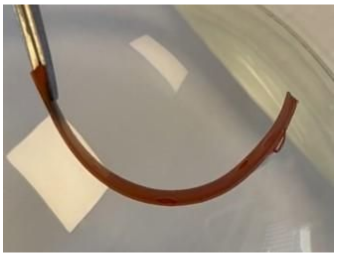	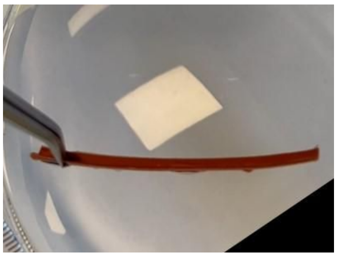
Aligned homogeneous film	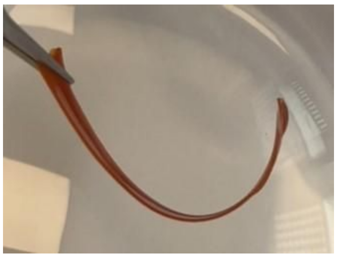	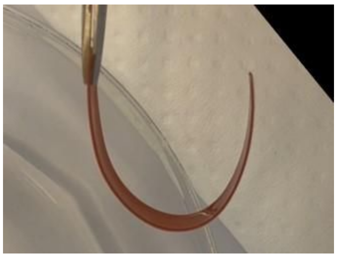	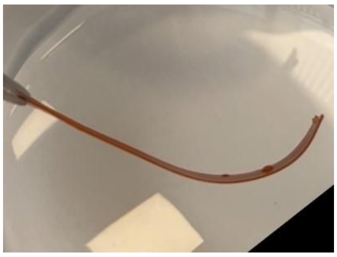
Unaligned homogeneous film	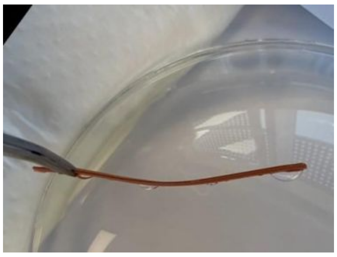	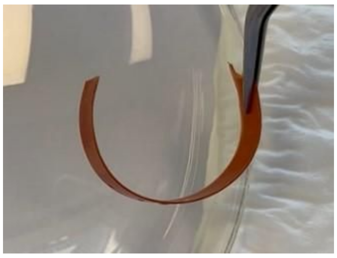	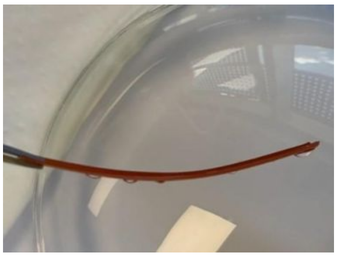

Pure polymer film	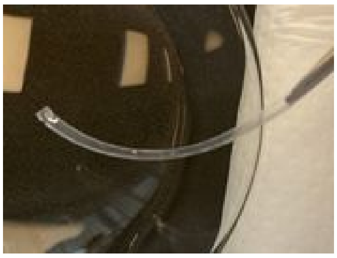	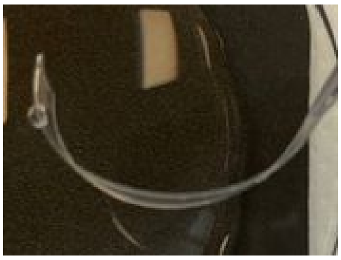	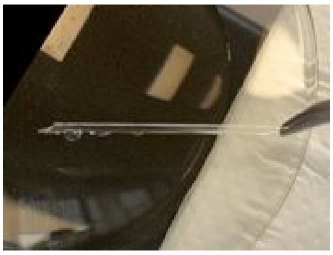

Table S3. Change of thickness of the small films between dry (in the presence of silica gel under mild vacuum), atmospheric and submerged conditions. Average value of three measurements, thickness is given in μm.

Film	Dry	Atmospheric	Submerged in water
Aligned gradient 1	129.0	138.7	146.0
Aligned gradient 2	131.0	138.0	148.7
Aligned gradient 3	135.3	137.0	153.0
Aligned gradient 4	132.3	142.7	149.0
Average aligned gradient	131.9	139.1	149.2
Unaligned gradient 1	134.0	138.7	148.3
Unaligned gradient 2	133.3	139.0	148.7
Unaligned gradient 3	135.0	139.3	151.0
Unaligned gradient 4	133.3	138.7	150.7
Average unaligned gradient	133.9	138.9	149.7

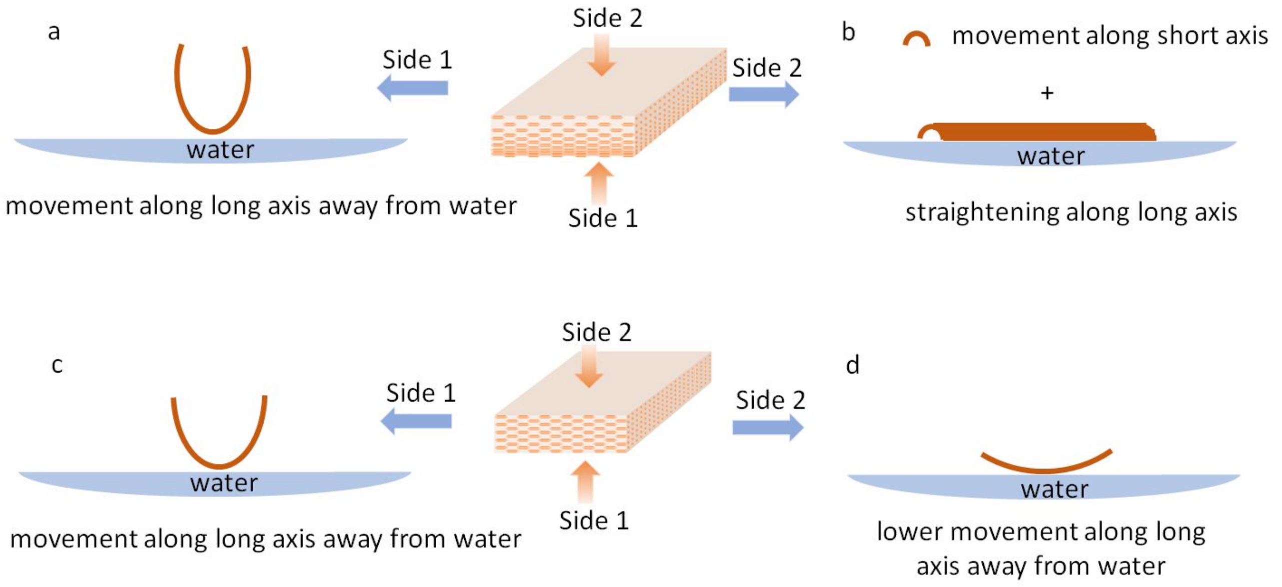

Figure S14. Directional movement of aligned films on the water surface depending on the side of the film exposed to water: films with gradient a) side 1, b) side 2 and homogeneous c) side 1, d) side 2.

Movement demonstrations are illustrated in supporting videos 1-4

SI video 1: Aligned gradient film behavior to water stimuli

SI video 2: Unaligned gradient film behavior to water stimuli

SI video 3: Aligned homogeneous film behavior to water stimuli

SI video 4: Unaligned homogeneous film behavior to water stimuli

3.2 Film response to the change of humidity

Different percentages of humidity were applied to the aligned and unaligned MIL-88A@PEGDA polymer films. It was found that the film curvature of aligned films increases along with the increase of humidity and vice versa. The overall degree of bending (□) is significantly larger in the aligned MIL-88A@PEGDA film compared to the unaligned film.

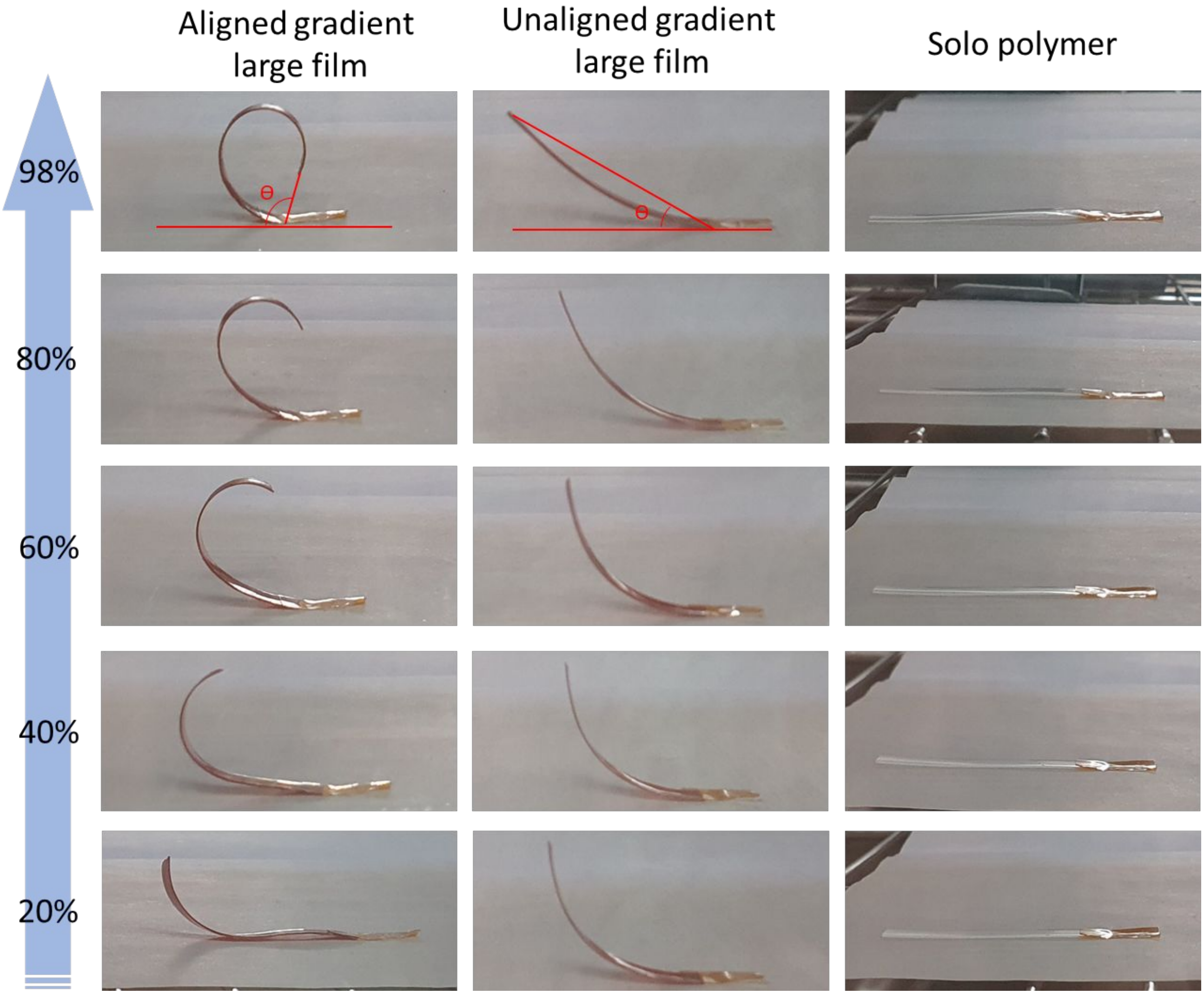

Figure S15. adsorption of water moisture at 20%, 40%, 60%, 80% and 98%

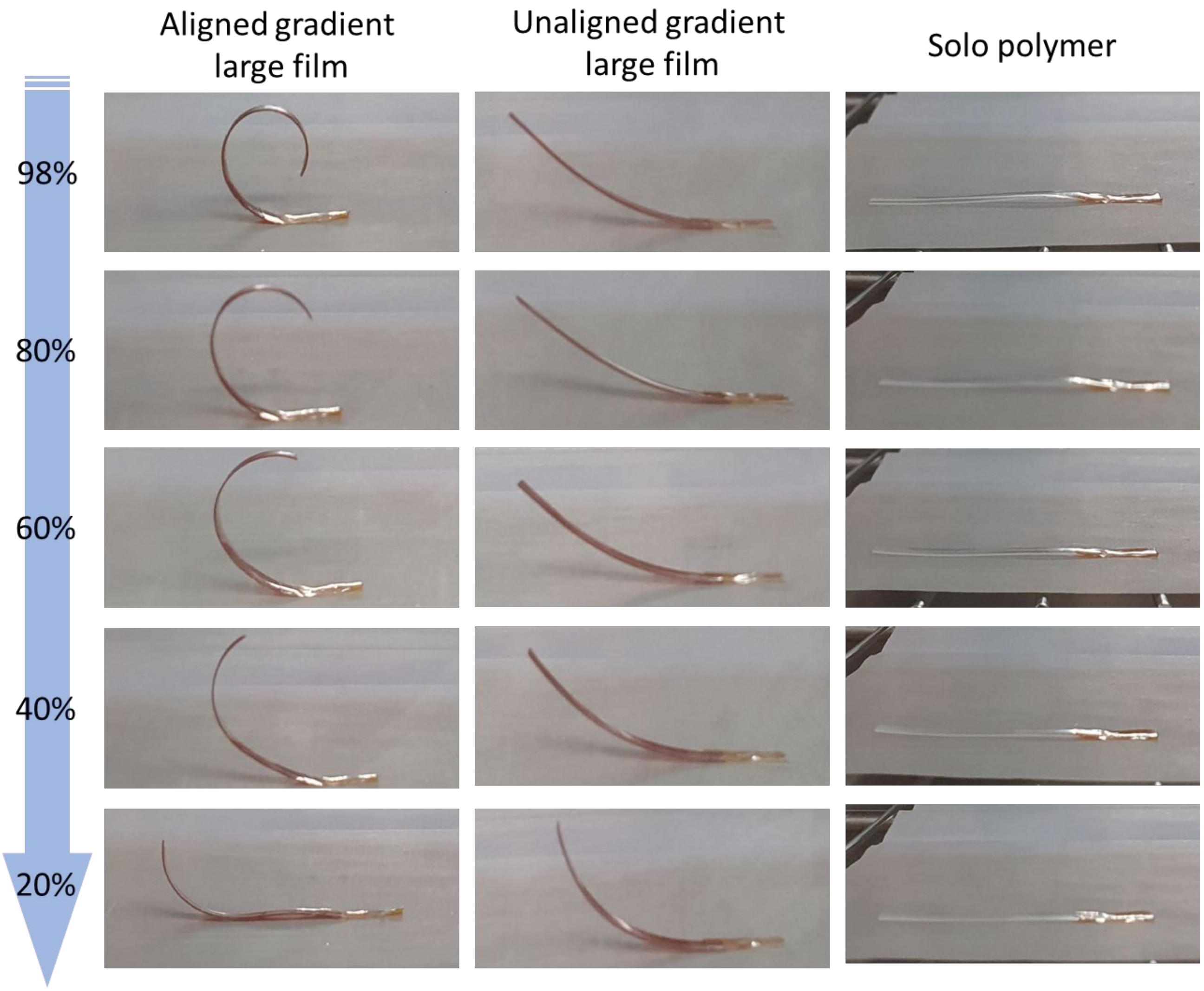

Figure S16. desorption of water moisture at 20%, 40%, 60%, 80% and 98%.

Table S4. degree of bending of the aligned MIL-88A@PEGDA versus unaligned MIL-88A@PEGDA large films at different humidity levels.

desorption						
		98%	80%	60%	40%	20%
Aligned gradient large film	C1	108.1	110.7	95	61.1	23.3
	C2	115.5	103.6	85.5	61.03	23.3
Unaligned gradient large film	C1	24.7	23.6	25.7	29.3	34.7
	C2	27.2	23.9	24.4	27.3	35.6
adsorption						
		20%	40%	60%	80%	98%
Aligned gradient large film	C1	29.8	39.6	75.4	96.3	108.1
	C2	23.3	50.6	77.1	88.8	115.5
Unaligned gradient large film	C1	67.03	59.5	52.2	40.7	24.7
	C2	39.9	43.8	42.6	38.8	27.2

C1: cycle 1, C2: cycle 2

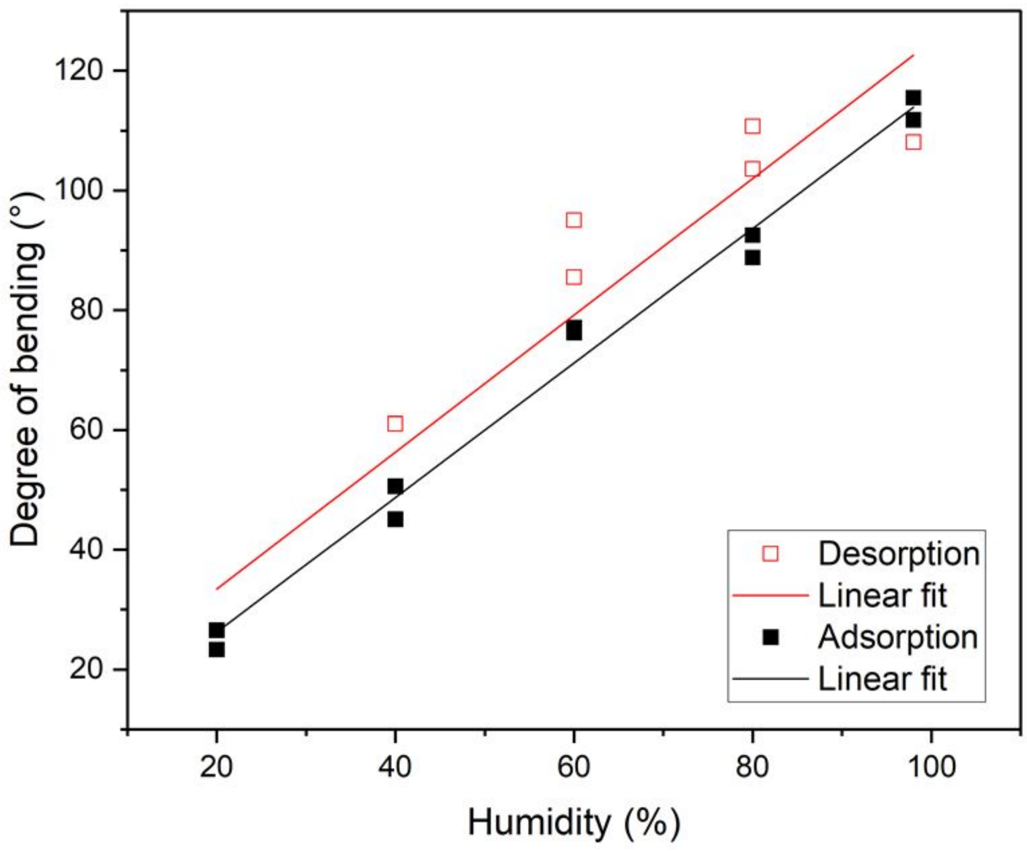

Figure S17. Degree of bending in aligned gradient large film versus change of humidity.

3.3 Directional movement of films with air stream

The directional movement of the films with humid/dry air flow was demonstrated with 20 cycles, switching between 1 minute with humid air and 1 minute with dry air to observe the bending behavior. Movements of the film are demonstrated in supporting video 5: Aligned gradient film cyclability to humid/dry air flow.

SI 4 Theoretical estimate

All calculations were made for the small film 21.4 mm × 3.0 mm × 0.13 mm.

The total % (*V*/ *V*) of MOF in the film was calculated using the theoretical density of MIL-88A crystal structure[9] (refcode FAVKAP, ρ=1.734 g/cm^3):

$$\%(V/V) = \frac{V\ (MIL\text{–}88A)}{V\ (film)} = \frac{m(MIL\text{–}88A)}{\rho(MIL88A)V(film)} = \frac{4}{1.734 \cdot 21.4 \cdot 3 \cdot 0.13} = 28\%$$

Assuming that all rod particles are ideally ordered and transfer their maximum 33% expansion to the film, the expected folding can be estimated from the difference of MOF content at the bottom and at the top of the film.

The vertical gradient in the film was estimated from the SEM cross section image (Figure S18) by calculating the percentage of particles in the polymer. We visually split the film into two layers (top and bottom, red line), converted the image to black and white and calculated the color profile in perpendicular direction (along green line).

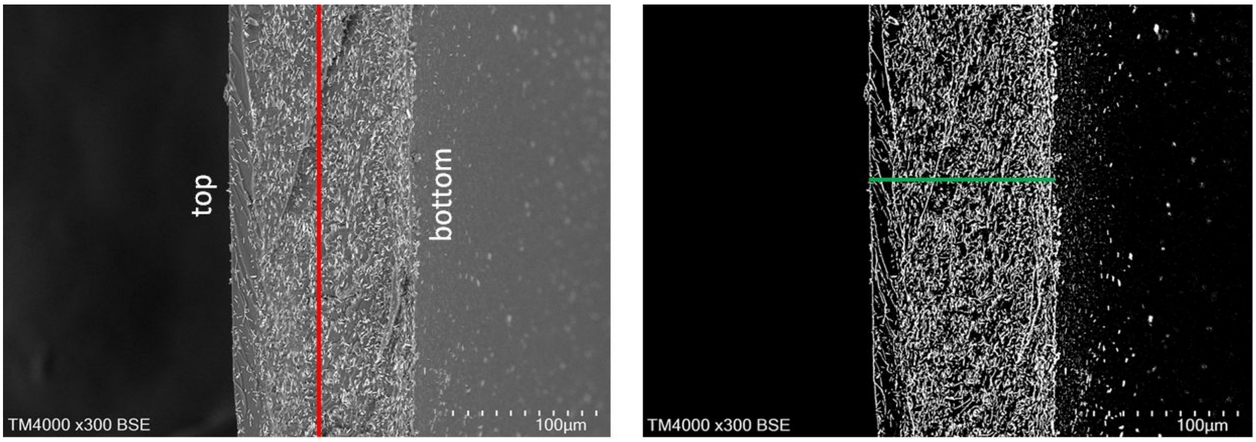

Figure S18. SEM image of gradient aligned small film used for theoretical estimate. The red line splits the film into top and bottom layers (on the left); the color profile of black and white image is calculated along the green line (on the right).

The number of white spots calculated for both layers lets us obtain the ratio between particles content in the top and bottom layers which is equal to 2/3. The average %(*V*/ *V*) in both layers was estimated from this ratio: top layer – 22.4% and bottom layer – 33.6% (average %(*V*/ *V*) = 28% calculated before).

The 22.4% content of particles in the top layer gives following: in 21.4 mm (length of the film) of the top layer there are 21.4 × 0.224 = 4.8 mm of MIL-88A particles which increase its width by 33% and make the top layer expand in water up to $l_1 = 21.4 + 4.8 \times 0.33 = 23.0$ mm. Analogous calculations for bottom layer give expansion to $l_2 = 21.4 + 21.4 \times 0.336 \times 0.33 = 23.8$ mm.

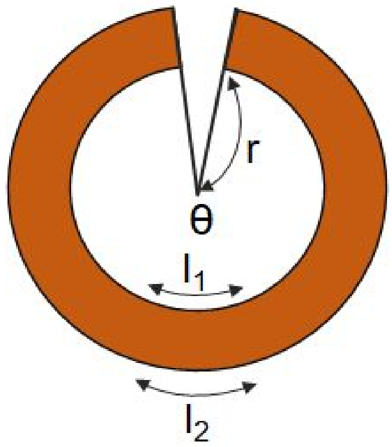

Figure S19. Theoretical estimate: schematic drawing of two arcs with radii r and (r+0.13), where 0.13 mm is the film thickness. Here $l_1 = \theta r = 23.0$ mm and $l_2 = \theta(r+0.13) = 23.8$ mm are the lengths of top and bottom layers of the film expanded in water.

$$\{\theta r = 23 \ \theta(r + 0.13) = 23.8$$

From the system of two equations it is easy to find that r = 3.7 mm and θ = 356˚. Thus, the angle of the film folding is very close to 360˚ which agrees very well with the observations.

SI 5 Film energy output

Film movement data and energy output calculations:

The movement of the film was measured in degrees (presented data are average of 3 measurements). For each weight, the film was allowed to relax at ambient humidity and the instrument was zeroed. The film was then subjected to a stream of dry air until stable, and the position recorded. Then the stream was switched to humid air and the instrument was again allowed to reach stability and the position recorded. Tweezers were gently positioned against the film to stabilize it during exposure to the air flow and retracted in order to read the movement. The angular movement was then transposed to linear motion with the conversion factor obtained previously (1° = 0.0178 mm), and the work was calculated following the standard formula of Newtonian physics.

Table S5. Movement data and energy output calculation

	Movement (°)		Movement (mm)			
Weight (mg)	wet	dry	wet	dry	sum	Work (µJ)
700	-105	31	1.87	0.55	2.42	16.61
925	-87	30	1.55	0.53	2.08	18.88
1025	-91	24	1.62	0.43	2.05	20.56

1250	-74	22	1.32	0.39	1.71	20.93
1575	-49	15	0.87	0.27	1.14	17.58
1985	-37	9	0.66	0.16	0.82	15.93
			**Average Energy (μJ)**		**σ (μJ)**	
			**18.42**		**2.06**	

Supporting video

SI video 6: Aligned gradient film used for measurements in dynamometer with humid/dry air flow

SI 6 References

(1) Pham, M. H.; Vuong, G. T.; Vu, A. T.; Do, T. O. Novel route to size-controlled Fe-MIL-88B-NH2 metal-organic framework nanocrystals. *Langmuir* **2011**, *27* (24), 15261-15267. DOI: 10.1021/la203570h

(2) Cheng, F.; Young, A. J.; Bouillard, J. G.; Kemp, N. T.; Guillet-Nicolas, R.; Hall, C. H.; Roberts, D.; Jaafar, A. H.; Adawi, A. M.; Kleitz, F.; et al. Dynamic Electric Field Alignment of Metal-Organic Framework Microrods. *J. Am. Chem. Soc.* **2019**, *141* (33), 12989-12993. DOI: 10.1021/jacs.9b06320

(3) Yanai, N.; Sindoro, M.; Yan, J.; Granick, S. Electric field-induced assembly of monodisperse polyhedral metal-organic framework crystals. *J. Am. Chem. Soc.* **2013**, *135* (1), 34-37. DOI: 10.1021/ja309361d

(4) Troyano, J.; Carne-Sanchez, A.; Maspoch, D. Programmable Self-Assembling 3D Architectures Generated by Patterning of Swellable MOF-Based Composite Films. *Adv. Mater.* **2019**, *31* (21), e1808235. DOI: 10.1002/adma.201808235

(5) Serre, C.; Mellot-Draznieks, C.; Surblé, S.; Auderbrand, N.; Filinchuk, Y.; Férey, G. Role of Solvent-Host Interactions that Lead to Very Large Swelling of Hybrid Frameworks. *Science* **2007**, *315* (5820), 1828-1831.

(6) Ren, G.; Zhao, K.; Zhao, L. A Fenton-like method using ZnO doped MIL-88A for degradation of methylene blue dyes. *RSC Adv.* **2020**, *10* (66), 39973-39980. DOI: 10.1039/d0ra08076d

(7) Liu, N.; Huang, W.; Zhang, X.; Tang, L.; Wang, L.; Wang, Y.; Wu, M. Ultrathin graphene oxide encapsulated in uniform MIL-88A(Fe) for enhanced visible light-driven photodegradation of RhB. *Appl. Catal., B* **2018**, *221*, 119-128. DOI: 10.1016/j.apcatb.2017.09.020.

(8) Horcajada, P.; Chalati, T.; Serre, C.; Gillet, B.; Sebrie, C.; Baati, T.; Eubank, J. F.; Heurtaux, D.; Clayette, P.; Kreuz, C.; et al. Porous metal-organic-framework nanoscale carriers as a potential platform for drug delivery and imaging. *Nat. Mater.* **2010**, *9* (2), 172-178. DOI: 10.1038/nmat2608

(9) Serre, C.; Millange, F.; Surblé, S.; Férey, G. A Route to the Synthesis of Trivalent Transition-Metal Porous Carboxylates with Trimeric Secondary Building Units. *Angew. Chem., Int. Ed.* **2004**, *43* (46), 6285-6289. DOI: 10.1002/anie.200454250.
